# CCR2 Is Dispensable for Disease Resolution but Required for the Restoration of Leukocyte Homeostasis Upon Experimental Malaria-Associated Acute Respiratory Distress Syndrome

**DOI:** 10.3389/fimmu.2020.628643

**Published:** 2021-02-16

**Authors:** Emilie Pollenus, Thao-Thy Pham, Leen Vandermosten, Queeny Robalo, Hendrik Possemiers, Sofie Knoops, Ghislain Opdenakker, Philippe E. Van den Steen

**Affiliations:** ^1^ Laboratory of Immunoparasitology, Department of Microbiology, Immunology and Transplantation, Rega Institute for Medical Research, KU Leuven, University of Leuven, Leuven, Belgium; ^2^ Laboratory of Immunobiology, Department of Microbiology, Immunology and Transplantation, Rega Institute for Medical Research, KU Leuven, University of Leuven, Leuven, Belgium

**Keywords:** malaria, inflammation, resolution, monocytes, immunology, parasitology, eosinophils

## Abstract

Malaria complications are often lethal, despite efficient killing of *Plasmodium* parasites with antimalarial drugs. This indicates the need to study the resolution and healing mechanisms involved in the recovery from these complications. *Plasmodium berghei* NK65-infected C57BL/6 mice develop malaria-associated acute respiratory distress syndrome (MA-ARDS) at 8 days post infection. Antimalarial treatment was started on this day and resulted in the recovery, as measured by the disappearance of the signs of pathology, in >80% of the mice. Therefore, this optimized model represents an asset in the study of mechanisms and leukocyte populations involved in the resolution of MA-ARDS. C-C chemokine receptor type 2 (CCR2) knock-out mice were used to investigate the role of monocytes and macrophages, since these cells are described to play an important role during the resolution of other inflammatory diseases. CCR2 deficiency was associated with significantly lower numbers of inflammatory monocytes in the lungs during infection and resolution and abolished the increase in non-classical monocytes during resolution. Surprisingly, CCR2 was dispensable for the development and the resolution of MA-ARDS, since no effect of the CCR2 knock-out was observed on any of the disease parameters. In contrast, the reappearance of eosinophils and interstitial macrophages during resolution was mitigated in the lungs of CCR2 knock-out mice. In conclusion, CCR2 is required for re-establishing the homeostasis of pulmonary leukocytes during recovery. Furthermore, the resolution of malaria-induced lung pathology is mediated by unknown CCR2-independent mechanisms.

## Introduction

Malaria is a global health disease that caused 229 million clinical cases and 409,000 deaths in 2019 (1). Most of these deaths are caused by complications, such as cerebral malaria, severe malarial anemia, placental malaria, and malaria-associated acute respiratory distress syndrome (MA-ARDS) (2). Despite efficient and rapid parasite killing with artemisinin combination therapies, 15% of patients with severe malaria still dies. Some complications, such as MA-ARDS, may even develop during or after treatment (3, 4). Artemisinins have a short half-life and act rapidly by generating free radicals inside the parasite (5). Artesunate is a highly soluble artemisinin derivative with excellent bioavailability, and is the preferred first-line treatment for severe malaria. It is often combined with other longer-acting antimalarial drugs, including quinolones such as chloroquine, which inhibit the detoxification of heme to hemozoin.

MA-ARDS mainly occurs in adults in low-transmission areas or in non-immune travellers (4). It results in a poor prognosis with mortality rates up to 80%, and it affects 2–25% of adults with severe malaria. Currently, 40% of patients with MA-ARDS succumb despite treatment with a combination of antimalarial drugs and mechanical ventilation. MA-ARDS is characterized by excessive pulmonary inflammation, resulting in the disruption of the alveolar-capillary membrane integrity and subsequent alveolar edema, microhemorrhages, and hypoxemia (2). An exaggerated Th1 immune response is proposed to be the cause of this complication (6–8). In mouse models, a pathogenic role of CD8^+^ T cells was identified, since depletion of CD8^+^ T cells resulted in the prevention of MA-ARDS (7, 9). In patients with MA-ARDS, lung endothelial cells are activated, resulting in the secretion of cytokines and chemokines and subsequent leukocyte accumulation in the lungs (7). In addition, the endothelial cells cross-present parasite antigens in an MHC-I context. Upon antigen recognition, CD8^+^ T cells produce interferon-γ (IFN-γ), granzyme B, and perforin, thereby causing apoptosis of endothelial cells and the breakdown of tight junctions of the alveolar-capillary membrane.

Resolution of inflammation is an active and coordinated process, which aims to restore normal functions of cells and tissues (10–12). During resolution, inflammatory cells such as neutrophils undergo apoptosis and are removed by phagocytosing macrophages, a process also known as efferocytosis (11). Wound healing mechanisms are crucial for the removal of debris and the restoration of tissue function. Monocytes and macrophages are thus suggested to play an important role during this resolution process (13). In the resolution process, monocytes may switch from an inflammatory phenotype (known as “M1” or Ly6C^+^) to a more reparative phenotype (known as “M2-like” or Ly6C^−^) or differentiate into macrophages or dendritic cells (DCs).

The study of resolution mechanisms is important, since new pro-resolving therapies might complement anti-inflammatory treatments (12, 14, 15). In fact, an adequate inflammatory response is often necessary for pathogen elimination and/or debris clearance. Pro-resolving approaches do not inhibit pathogen clearance in contrast to anti-inflammatory treatments. Pro-resolving agonists have a more broad mechanism of action involving modulation of the immune response and stimulation of repair, while anti-inflammatory molecules strongly inhibit the immune response (14, 15). Therefore, it is assumed that pro-resolving therapies are able to suppress inflammation with less unwanted side-effects, in comparison with anti-inflammatory treatments. At the same time, resolution processes promote the restoration of tissue function (14). Resolution of inflammation has been well-characterized in Th2-related diseases, such as helminth infections. Induction of the “M2-like” macrophage phenotype by interleukin-(IL-)4 and IL-13 does not only promote the control of helminth infection, but also the timely conversion of monocytes and macrophages from a pro-inflammatory “M1” to a reparative “M2-like” phenotype, which might be decisive in wound healing and tissue regeneration (16–19).

In contrast, much less is known about the resolution of Th1-related inflammation, in particular in malaria. The resolution mechanisms may be different in a Th1 *versus* a Th2-related disease. Only a few studies investigated the pro-resolving effects of exogenous molecules, such as lipoxin A4 and IL-33, in malaria (20–24). However, the complete resolution process and the endogenous players involved have not yet been studied in detail.

C-C chemokine receptor type 2 (CCR2) is crucial for the trafficking of monocytes in two ways (25). First, CCR2 is involved in the homeostatic release of monocytes from the bone marrow. Secondly, CCR2^+^ Ly6C^+^ inflammatory monocytes (iMOs) are recruited to the site of inflammation *via* the CCL2-CCR2 axis (25, 26). Therefore, CCR2 knock-out (KO) mice have lower numbers of circulating monocytes and are often used to study the roles of monocytes *in vivo*. In the development of MA-ARDS, the effect of CCR2 KO is limited (8, 27). In *Plasmodium berghei* ANKA-infected C57BL/6 mice, the wet:dry ratio of the lungs was slightly increased in CCR2 KO mice compared to wild-type (WT) mice, whereas no effect on alveolar edema was found (27). Also in *Plasmodium berghei* NK65 (*Pb*NK65)-infected C57BL/6 mice, no difference in survival between the CCR2 WT and KO mice was found (8). Despite the limited effect on the development of pathology, the effect of CCR2 on the resolution remains to be established. Interestingly, CCR2 was shown to be crucial for debris clearance and healing in a sterile thermal liver injury and a skin wound mouse model (28, 29).

Here, we adapted our mouse model to study the resolution of inflammation in the otherwise lethal MA-ARDS complication. Starting antimalarial treatment on the day that the first clinical disease symptoms appeared in *Pb*NK65-infected C57BL/6 mice, resulted in the efficient clearance of parasites followed by resolution of the inflammatory lung pathology. As 80% of the mice were rescued, this constitutes an excellent mouse model to study the mechanisms and leukocytes involved in the recovery from MA-ARDS. In addition, CCR2 KO mice were used to investigate the role of CCR2-dependent monocytes in the development and the resolution of malaria-induced lung pathology. Our data show that, although the CCR2 KO has no effect on the development nor the resolution of MA-ARDS, CCR2 is crucial to re-establish the homeostasis of pulmonary leukocytes, since the reappearance of eosinophils and interstitial macrophages was mitigated in the lungs of *Pb*NK65-infected CCR2 KO mice.

## Materials and Methods

### Mice and Dissections

Seven to eight weeks old C57BL/6 mice were purchased from Janvier Labs (Le Genest-Saint-Isle, France) and housed in a specific pathogen-free (SPF) facility. Seven to nine weeks old SPF CCR2 KO and CCR2 WT mice were bred in the animal house of the Rega Institute for Medical Research, KU Leuven. CCR2 KO mice were originally bought from The Jackson Laboratory (B6.129S4-Ccr2tm1Ifc/J; #004999; Bar Harbor, ME, USA) and C57BL/6J mice from Charles River (JAX™ C57BL/6J SOPF Mice; #680; Lyon, France). The CCR2 KO mice were mated with the C57BL/6J mice in order to generate F1 heterozygotes. These heterozygotes were inter-crossed to create CCR2 KO and matched CCR2 WT mice. All mice were housed in individually ventilated cages and received *ad libitum* high energy food (Ssniff Spezialdiäte GMBH, Soest, Germany) and water, which was supplemented with 0.422 mg/ml 4-amino-benzoic acid sodium (PABA; Sigma-Aldrich, Bornem, Belgium). All experiments were performed at the KU Leuven according to the regulations of the European Union (directive 2010/63/EU) and the Belgian Royal Decree of 29 May 2013, and were approved by the Animal Ethics Committee of the KU Leuven (License LA1210186, project P049/2018, Belgium). Mice were euthanized by intraperitoneal (i.p.) injection of 100 µl of dolethal (Vétoquinol, Aartselaar, Belgium; 200 mg/ml). Murine blood samples were obtained by cardiac puncture in heparinized (LEO, Pharma, Lier, Belgium) syringes. To obtain broncho-alveolar lavage fluid (BALF), 500 µl or 750 µl of Dulbecco’s phosphate buffered saline was instilled through a catheter in the trachea in the right lungs after pinching off the left lung, or the complete lungs, respectively. After 30s the fluid was withdrawn. This was repeated and both lavages were pooled. The BALF was centrifuged (10 min, 314 g, 4°C) and the supernatant was collected for further analysis, whereas the cell pellet was combined with the cells isolated from the lungs for flow cytometry analysis. After transcardial perfusion, lungs and spleen were collected for analysis by flow cytometry or reverse transcriptase quantitative real time polymerase chain reaction (RT-qPCR).

### Genotyping

DNA was isolated from the tails or ear snippets of CCR2 KO and CCR2 WT mice using the EZNA Tissue DNA Kit (Omega Bio-Tek, Norcross, GA, USA). PCR was performed followed by gel electrophoresis to confirm the CCR2 genotypes, according to the protocol of The Jackson Laboratory (WT primers: CCA CAG AAT CAA AGG AAA TGG and CAC AGC ATG AAC AAT AGC CAA G, KO primers: CCA CAG AAT CAA AGG AAA TGG and CCT TCT ATC GCC TTC TTG ACG). In order to confirm the effective C57BL/6J background in both the CCR2 KO and WT mice, background strain characterization through genome-wide SNP analysis was performed on tail or ear genomic DNA from two original CCR2 KO mice and two original C57BL/6J mice and from three CCR2 KO and 3 CCR2 WT mice after the backcross (Mouse Genome Scanning panel of 2050 SNPs, Taconic, Rensselaer, NY, USA).

### Infection of Mice and Clinical Scoring

Mice were infected with *Pb*NK65 [Edinburgh strain (30, 31)] by i.p. injection of 10^4^ infected red blood cells. Non-infected controls from the same sex and age were included in each experiment. The disease severity of the mice was evaluated based on body weight, parasitemia, and clinical score. The clinical score was calculated on the basis of different parameters: social activity (SA), limb grasping (LG), body tone (BT), trunk curl (TC), pilo-erection (PE), shivering (Sh), abnormal breathing (AB), dehydration (D), incontinence (I), and paralysis (P). In case of TC, PE, Sh and AB, a disease score of 0 (absent) or 1 (present) was given, while the other parameters received a score of 0 (normal), 1 (intermediate) or 2 (serious). The formula used to calculate the total clinical score was: SA + LG + BT + TC + PE + 3*(Sh + AB + D + I + P). Parasitemia was determined by staining blood smears with 10% Giemsa’s Stain Improved R66 Solution (VWR, Heverlee, Belgium).

### Antimalarial Treatment

Where indicated, mice were treated with antimalarial drugs. A combination of artesunate (ART, 10 mg/kg in 0.9% NaCl with 0.1% NaHCO_3_; Sigma-Aldrich) and chloroquine diphosphate salt (CQ, 30 mg/kg in 0.9% NaCl; Sigma-Aldrich) was i.p. injected daily in a volume of 200 µl, starting at 8 days p.i. for a maximum of 5 days. In the experiments described in **Supplementary Figure 1**, a higher dose of 40 mg/kg of ART (without CQ) and a combination of 10 mg/kg of ART with 3 mg/kg of dexamethasone sodium phosphate (in 0.9% NaCl; Sigma-Aldrich) were also used.

### Quantification of BALF protein concentration

Edema formation was assessed by determination of the protein concentration in the supernatant of the BALF samples using Bradford assay (Bio-Rad, Hercules, CA, USA).

### Determination of mRNA Expression Levels

mRNA expression levels in left lungs were quantified with RT-qPCR. Therefore, RNA extraction was performed on the left lung using the QIAGEN’s RNeasy Mini Kit (QIAGEN, Venlo, The Netherlands) according to the manufacturer’s protocol. Next, cDNA was synthesized from the extracted RNA using the High Capacity cDNA Reverse Transcription Kit (Applied Biosystems, Life Technologies). The TaqMan^®^ Fast Universal PCR master mix (Applied Biosystems) was used for the detection of the amplification of the targeted gene in combination with specific primers (**Supplementary Table 1**). The relative mRNA expression was determined using the 2^−ΔΔCt^ method (32), which reflected the fold change in gene expression compared to the mean of the uninfected controls and was further normalized to the 18S housekeeping gene.

### Isolation of Leukocytes From the Spleen

During dissection, spleens were removed and collected in phosphate buffered saline (PBS) + 2% fetal calf serum (FCS, Gibco) at 4°C. Single cells were obtained after mashing the spleen through a 70 µm nylon cell strainer (VWR) followed by treatment with a RBC lysis buffer (0.83% ammonium chloride (NH_4_Cl; Acros Organics, Geel, Belgium)/10 mM Tris (Sigma) solution with pH 7.2) at 37°C. After washing with PBS + 2% FCS, cells were resuspended in PBS + 2% FCS and live leukocytes were counted in a Bürker chamber after 1/2 dilution in trypan blue (VWR).

### Isolation of Leukocytes From the Lungs

#### Protocol 1

During dissection, lungs were removed and collected in HEPES buffer (10 mM HEPES-NaOH, 150 mM NaCl, 5 mM KCl, 1 mM MgCl_2_, 1.8 mM CaCl_2_, pH 7.4) at room temperature (RT) in gentle MACS C tubes (MACS Miltenyi Biotec, Leiden, The Netherlands). The lungs were homogenized in 5 ml of HEPES buffer with 2 mg/ml collagenase D (Sigma-Aldrich) and 0.04 mg/ml DNase I (Sigma-Aldrich) in the gentleMACS™ Dissociator (MACS Miltenyi Biotec) followed by incubation for 30 min at 37°C. After a second processing in the gentleMACS™ Dissociator, cells were passed through a 70 µm nylon cell strainer. After centrifugation (300 g, 7 min, RT), the non-leukocyte subsets were removed with a Percoll gradient [for 100% Percoll buffer: 90% Percoll (GE Healthcare, Upsala, Sweden)], 9 mM PBS, 0.01 M HEPES (Gibco, Thermo Fischer), and 0.005 N HCl). The cell pellet was resuspended in 40% Percoll buffer in PBS and dropwise added onto 72% Percoll buffer in PBS to get two distinct layers. After centrifugation (20 min, 491 g, RT, no break), leukocytes were collected from the interphase of the two Percoll layers. After washing with PBS + 2% FCS, cells were resuspended in PBS + 2% FCS and live leukocytes were counted in a Bürker chamber after 1/2 dilution in trypan blue.

#### Protocol 2

During dissection, lungs were removed and collected in RPMI buffer [RPMI GlutaMAX (Gibco) + 5% FCS + 1% penicillin/streptomycin (Gibco)] with 0.1% beta-mercaptoethanol at RT. Lungs were first minced with scissors and then incubated for 30 min at 37°C in digestion medium (2 mg/ml collagenase D and 0.1 mg/ml DNase I in RPMI buffer). Afterward, pieces of tissue were homogenized using a needle and syringe and fresh digestion medium was added for a second incubation at 37°C for 15 min. Lung tissue was homogenized again with the syringe and centrifuged (5 min, 708 g, RT). The cell pellet was resuspended using 10 mM EDTA and further diluted in PBS + 2% FCS. After a second centrifugation, cells were treated with 0.83% NH_4_Cl/10 mM Tris to lyse RBCs at 37°C and passed through a 70 µm nylon cell strainer. Cells were washed and resuspended in PBS + 2% FCS and live leukocytes were counted in a Bürker chamber after 1/2 dilution in trypan blue.

### Staining and Flow Cytometry of Leukocytes

1.5–3 million leukocytes per sample were transferred to 96 well plates and washed with PBS. Cells were incubated for 15 min at RT in the dark with a viability dye, Zombie Aqua (1/1,000; BioLegend, San Diego, CA, USA) or Zombie UV (1/1,000; BioLegend), together with Mouse Fc block (MACS Miltenyi Biotec). After washing twice with cold PBS + 2% FCS + 2 mM EDTA, the cells were stained with a panel of monoclonal antibodies (**Supplementary Table 2**) dissolved in Brilliant stain buffer (BD Biosciences; Erembodegem, Belgium) for 20 min at 4°C in the dark. Cells were transferred to FACS tubes, washed twice with PBS, and fixed in PBS + 0.4% formaldehyde.

100,000 or 200,000 live single cells were analyzed per sample with a BD LSR Fortessa Flow cytometer (BD Biosciences), depending on the panel (**Supplementary Table 2**). Data were analyzed with FlowJo v10 software (FlowJo LLC, Ashland, OR, USA) and cells were gated according to the gating strategies in supplementary methods. In the myeloid cell panel and the CCR2 panel, cells positive for lineage-specific markers were excluded using a dumpgate (with CD3, CD19, and NK1.1 as exclusion markers). In order to calculate the absolute numbers of each cell type, the frequency of this cell type among CD45^+^ cells was multiplied by the total number of live leukocytes earlier counted using the Bürker chamber. In addition, uniform manifold approximation and projection (UMAP) plots were created of the flow cytometry data of the myeloid panel on the lungs. Here, cells of different samples were combined and unbiased clustering was performed using the FlowJo v10 software. Clusters were colored by cell class as defined in the figure legends.

### Statistical Analysis

**Figure 1:**
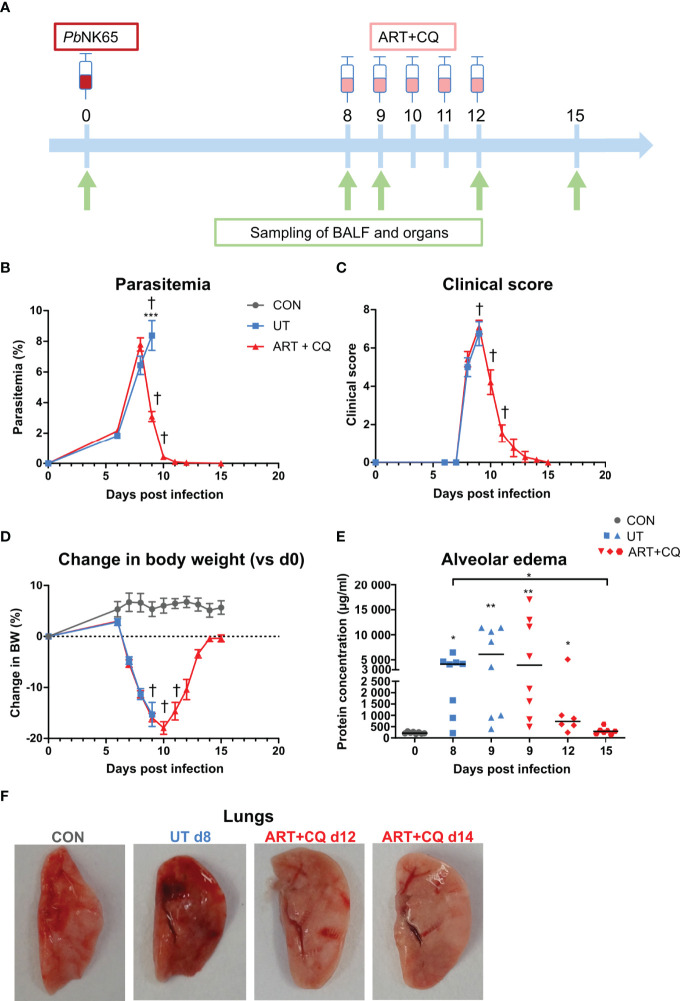
A mouse model to study the resolution of malaria-associated acute respiratory distress syndrome (MA-ARDS) after antimalarial treatment. C57BL/6 mice were infected with *Pb*NK65. Mice were injected daily from 8 until 12 days p.i. with 10 mg/kg ART + 30 mg/kg CQ (ART+CQ). **(A)** Schematic representation of the timing of infection and antimalarial treatments in the mouse model. **(B)** Parasitemia was determined using Giemsa-stained blood smears. **(C)** The clinical score was monitored daily starting at 6 days p.i. **(D)** The change in body weight was calculated compared to day 0 p.i. starting at 6 days p.i. **(B, D)** Compilation of two experiments. Data are means ± SEM. n=8 for uninfected controls (CON), n=8–16 for the infected untreated group (UT), n=7–21 for the infected ART+CQ-treated group. **(E)** The protein concentration in the BALF was determined as a measure of alveolar edema. Compilation of two experiments. Each symbol represents data of an individual mouse. n=8 for CON on day 0, UT at 8 and 9 days p.i and ART+CQ at 9 days p.i., n=6 for ART+CQ at 12 days p.i., n=7 for ART+CQ at 15 days p.i. **(F)** Representative pictures of the left lung.

The data were analyzed using the GraphPad PRISM software (GraphPad, San Diego, California, USA, version 8.3.1). The non-parametric Mann-Whitney U test was performed followed by the Holm-Bonferroni correction. For the experiments in **Figures 1** **–** **5** and **Supplementary Figures 1**-**5**, significance was determined for the comparison of all groups compared to the uninfected controls at 0 days p.i. and of all infected groups from 9 days until 15 days p.i. compared to the untreated mice at 8 days. In the experiments with CCR2 KO and CCR2 WT mice (**Figures 6** **–** **9** and **Supplementary Figures 7**-**16**), significance was determined between each condition for the CCR2 WT and for the CCR2 KO mice and between the WT and KO mice within each condition. P-values were indicated as follows: *p<0.05, **p<0.01, ***p<0.001. The horizontal black line in each group indicates the median. Statistical differences compared to the corresponding uninfected control group are indicated with an asterisk above the individual data sets and horizontal lines with asterisk on top indicate significant differences between groups.

**Figure 2:**
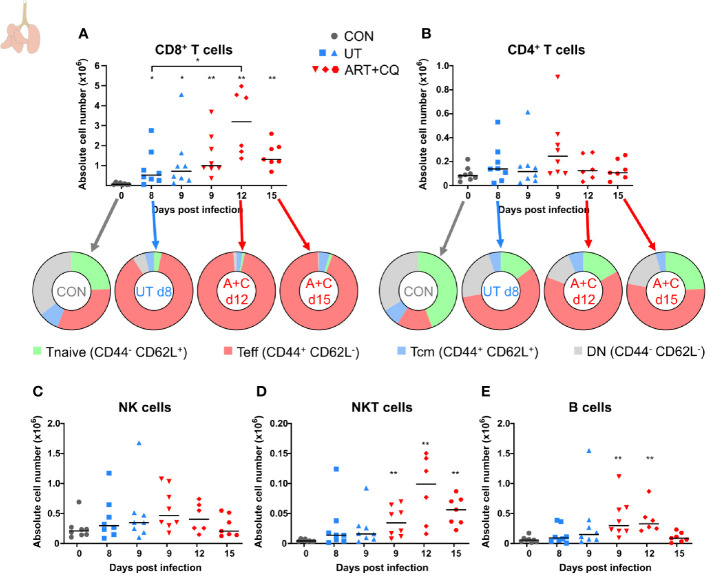
Dynamics of the lymphocyte populations in the lungs during resolution of malaria-associated acute respiratory distress syndrome (MA-ARDS). C57BL/6 mice were infected with *Pb*NK65. Mice were injected daily from 8 until 12 days p.i. with 10 mg/kg ART + 30 mg/kg CQ. Mice were dissected at the indicated days p.i. Leukocytes were isolated from the lungs according to protocol 1 and flow cytometry was performed. **(A, B)** The absolute numbers of CD8^+^ T cells (CD45^+^ CD3^+^ NK1.1^−^ CD8^+^) and CD4^+^ T cells (CD45^+^ CD3^+^ NK1.1^−^ CD4^+^) and the proportions of Tnaive (CD44^−^ CD62L^+^), Teff (CD44^+^ CD62L^−^), and Tcm (CD44^+^ CD62L^+^) of the total cell population are shown. Percentages of these subsets are shown in **Supplementary Figure 3**. **(C–E)** The absolute numbers of NK cells (CD45^+^ CD3^−^ NK1.1^+^), NKT cells (CD45^+^ CD3^+^ NK1.1^+^), and B cells (CD45^+^ CD3^−^ NK1.1^−^ B220^+^) were calculated. Compilation of two experiments. Each symbol represents data of an individual mouse. n=8 for CON on day 0, UT at 8 and 9 days p.i and ART+CQ at 9 days p.i., n=6 for ART+CQ at 12 days p.i., n=7 for ART+CQ at 15 days p.i.

**Figure 3:**
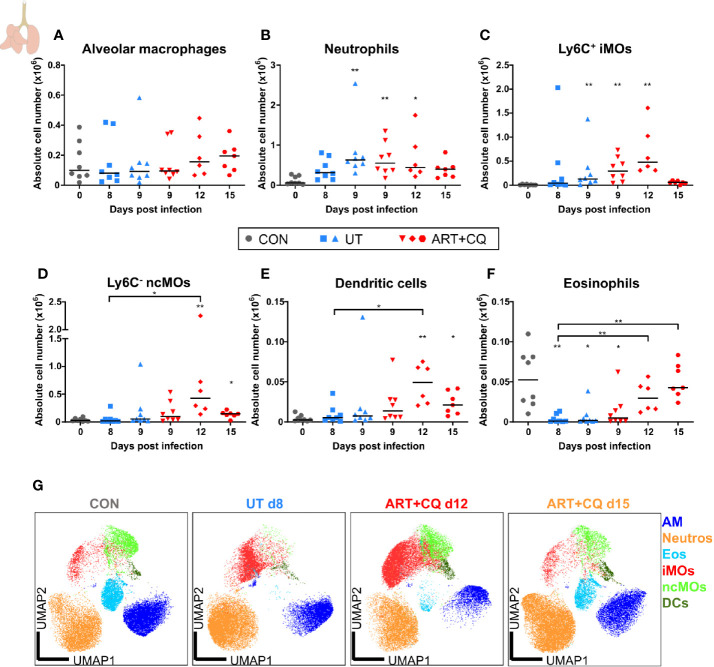
Dynamics of the myeloid cell populations in the lungs during resolution of malaria-associated acute respiratory distress syndrome (MA-ARDS). C57BL/6 mice were infected with *Pb*NK65. Mice were injected daily from 8 until 12 days p.i. with 10 mg/kg ART + 30 mg/kg CQ. Mice were dissected at the indicated days p.i. Leukocytes were isolated from the lungs according to protocol 1 and flow cytometry was performed. **(A–F)** The absolute numbers of alveolar macrophages (AM; CD45^+^ SiglecF^+^ CD11b^int^ CD11c^+^), neutrophils (Neutros; CD45^+^ Lin^−^ CD11b^+^ Ly6G^+^), Ly6C^+^ inflammatory monocytes (iMOs; CD45^+^ Lin^−^ CD11b^hi^ MHC-II^−^ Ly6C^+^), Ly6C^−^ non-classical monocytes (ncMOs; CD45^+^ Lin^−^ CD11b^hi^ MHC-II^−^ Ly6C^−^), dendritic cells (DCs; CD45^+^ Lin^−^ SiglecF^−^ MHC-II^+^ CD11c^+^), and eosinophils (Eos; CD45^+^ CD11b^+^ SiglecF^+^ CD11c^−^) in the lungs were calculated. Compilation of two experiments. Each symbol represents data of an individual mouse. n=8 for CON on day 0, UT at 8 and 9 days p.i and ART+CQ at 9 days p.i., n=6 for ART+CQ at 12 days p.i., n=7 for ART+CQ at 15 days p.i. **(G)** Clustering of 24,000 cells combined from four representative samples per condition, except ART+CQ d15 from two representative samples. The plots show a two-dimensional representation (UMAP) of the protein expression. Clusters are colored by cell class as defined in **(A–F)**.

**Figure 4:**
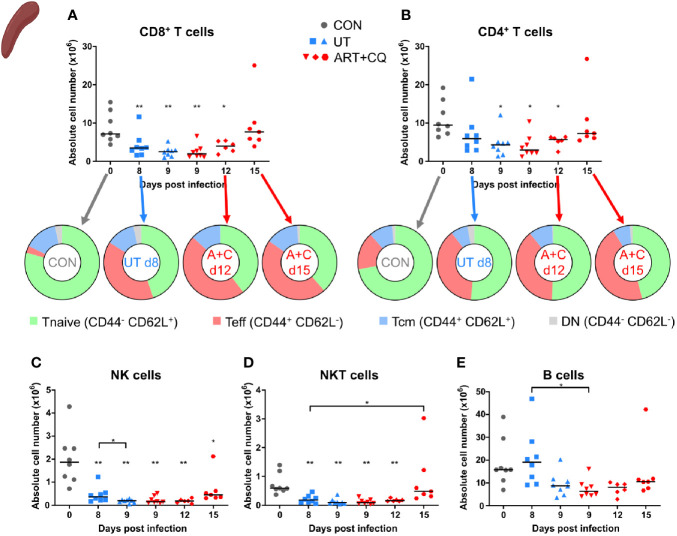
Dynamics of the splenic lymphocyte populations during resolution of malaria-associated acute respiratory distress syndrome (MA-ARDS). C57BL/6 mice were infected with *Pb*NK65. Mice were injected daily from 8 until 12 days p.i. with 10 mg/kg ART + 30 mg/kg CQ. Mice were dissected at the indicated days p.i. Leukocytes were isolated from the spleen and flow cytometry was performed. **(A, B)** The absolute numbers of CD8^+^ T cells (CD45^+^ CD3^+^ NK1.1^−^ CD8^+^) and CD4^+^ T cells (CD45^+^ CD3^+^ NK1.1^−^ CD4^+^) and the proportions of Tnaive (CD44^−^ CD62L^+^), Teff (CD44^+^ CD62L^−^), and Tcm (CD44^+^ CD62L^+^) of the total cell population are shown. Percentages of these subsets are shown in **Supplementary Figure 5**. **(C–E)** The absolute numbers of NK cells (CD45^+^ CD3^−^ NK1.1^+^), NKT cells (CD45^+^ CD3^+^ NK1.1^+^), and B cells (CD45^+^ CD3^−^ NK1.1^−^ B220^+^) were calculated. Compilation of two experiments. Each symbol represents data of an individual mouse. n=8 for CON on day 0, UT at 8 and 9 days p.i and ART+CQ at 9 days p.i., n=6 for ART+CQ at 12 days p.i., n=7 for ART+CQ at 15 days p.i.

**Figure 5:**
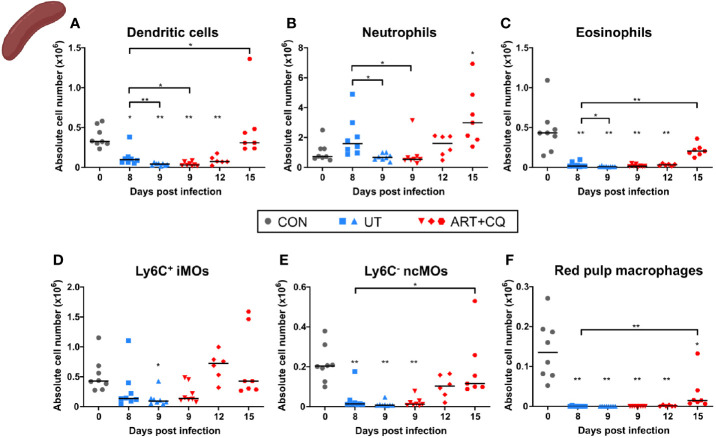
Dynamics of the splenic myeloid cell populations during resolution of malaria-associated acute respiratory distress syndrome (MA-ARDS). C57BL/6 mice were infected with *Pb*NK65. Mice were injected daily from 8 until 12 days p.i. with 10 mg/kg ART + 30 mg/kg CQ. Mice were dissected at the indicated days p.i. Leukocytes were isolated from the spleen and flow cytometry was performed. **(A–F)** The absolute numbers of dendritic cells (CD45^+^ Lin^−^ MHC-II^+^ CD11c^+^), neutrophils (CD45^+^ Lin^−^ CD11b^+^ Ly6G^+^), eosinophils (CD45^+^ CD11b^+^ MHC-II^−^ CD11c^−^ Ly6G^−^ Ly6C^−^ SSC-A^hi^), Ly6C^+^ inflammatory monocytes (iMOs; CD45^+^ Lin^−^ CD11b^+^ MHC-II^−^ CD11c^−^ SSC-A^lo^ Ly6C^+^), Ly6C^−^ non-classical monocytes (ncMOs; CD45^+^ Lin^−^ CD11b^+^ MHC-II^−^ CD11c^−^ SSC-A^lo^ Ly6C^−^), and red pulp macrophages [CD45^+^ Lin^−^ MHC-II^−^ CD11c^−^ CD11b^−^ F4/80^+^ (33)] present in the spleen were calculated. Compilation of two experiments. Each symbol represents data of an individual mouse. n=8 for CON on day 0, UT at 8 and 9 days p.i and ART+CQ at 9 days p.i., n=6 for ART+CQ at 12 days p.i., n=7 for ART+CQ at 15 days p.i.

**Figure 6:**
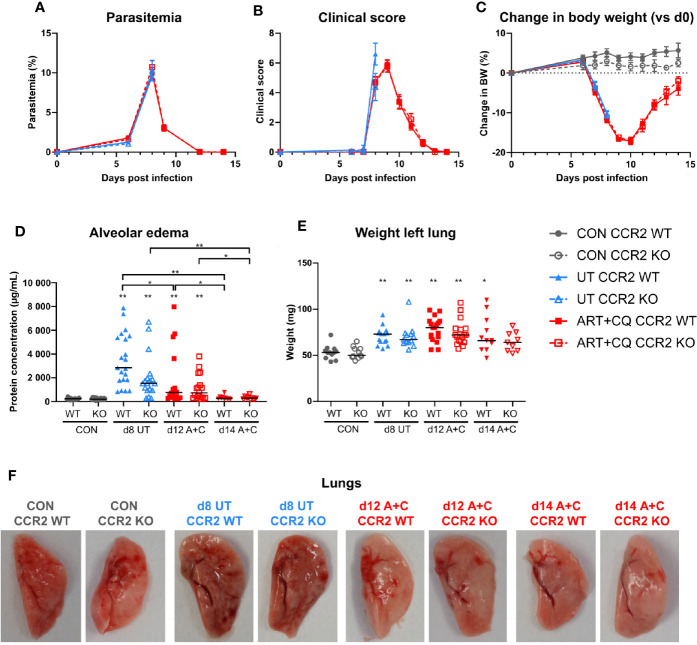
The CCR2 knock-out had no effect on the development and the resolution of malaria-associated acute respiratory distress syndrome (MA-ARDS). CCR2 WT and CCR2 KO C57BL/6 mice were infected with *Pb*NK65. Mice were injected daily from 8 until 12 days p.i. with 10 mg/kg ART + 30 mg/kg CQ. **(A)** Parasitemia was determined daily using Giemsa-stained blood smears. **(B)** The clinical score was monitored daily starting at 6 days p.i. **(C)** The change in body weight was calculated compared to day 0 p.i. starting at 6 days p.i. **(A–C)** Compilation of five experiments. Data are shown as means ± SEM. n=6–12 for CON CCR2 WT, n=5–11 for CON CCR2 KO, n=15 for UT CCR2 WT, n=13 for UT CCR2 KO, n=10–32 for ART+CQ CCR2 WT, n=7–27 for ART+CQ CCR2 KO. **(D, E)** Lung pathology was quantified based on the protein concentration in the BALF **(D)** and the weight of the left lung **(E)** at 8 days p.i. for the UT group and at 12 and 14 days p.i. for the ART+CQ group. Compilation of five experiments. Each symbol represents data of an individual mouse. n=12–16 for CON CCR2 WT, n=11–15 for CON CCR2 KO, n=15–21 for UT CCR2 WT, n=13–18 for UT CCR2 KO, n=18 for ART+CQ CCR2 WT at 12 days p.i., n=17 for ART+CQ CCR2 KO at 12 days p.i., n=10–11 for ART+CQ CCR2 WT at 14 days p.i., n=9 for ART+CQ CCR2 KO at 14 days p.i. **(F)** Representative pictures of the left lung.

**Figure 7:**
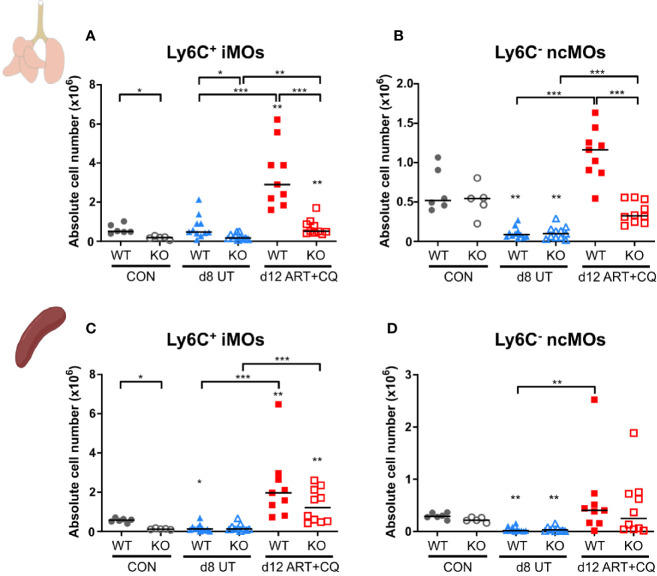
The CCR2 knock-out resulted in less monocytes present in the lungs, but not in the spleen. CCR2 WT and CCR2 KO C57BL/6 mice were infected with *Pb*NK65. Mice were injected daily from 8 until 12 days p.i. with 10 mg/kg ART + 30 mg/kg CQ. Mice were dissected at the indicated days p.i. Leukocytes were isolated from the lungs according to protocol 2 and from the spleen and flow cytometry was performed. **(A–D)** The absolute numbers of Ly6C^+^ iMOs **(A, C)** and Ly6C^−^ ncMOs **(B, D)** present in the lungs **(A, B)** and spleen **(C, D)** were calculated. Compilation of two experiments. Each symbol represents data of an individual mouse. n=6 for CON CCR2 WT, n=5 for CON CCR2 KO, n=11 for UT CCR2 WT, n=11 for UT CCR2 KO, n=9 for ART+CQ CCR2 WT, n=10 for ART+CQ CCR2 KO.

**Figure 8:**
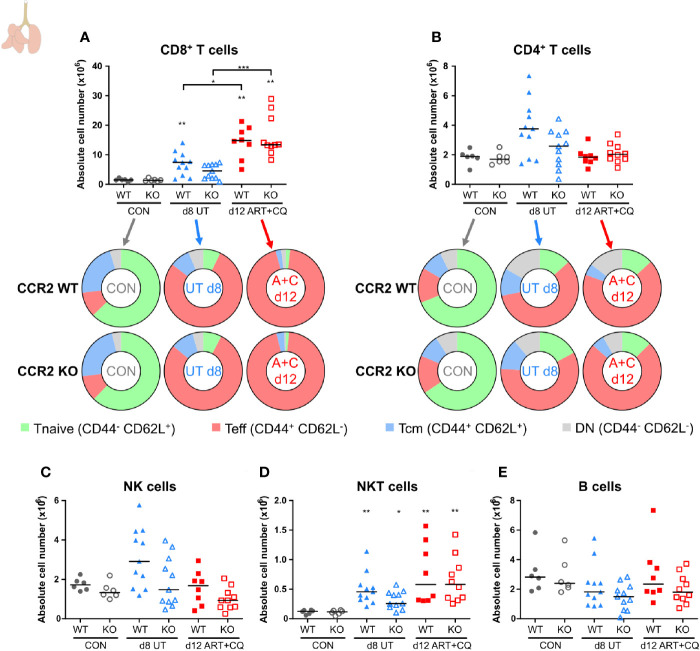
CCR2 knock-out does not affect the number of pulmonary lymphocytes. CCR2 WT and CCR2 KO C57BL/6 mice were infected with *Pb*NK65. Mice were injected daily from 8 until 12 days p.i. with 10 mg/kg ART + 30 mg/kg CQ. Mice were dissected at the indicated days p.i. Leukocytes were isolated from the lungs according to protocol 2 and flow cytometry was performed. **(A, B)** The absolute numbers of CD8^+^ T cells (CD45^+^ CD3^+^ NK1.1^−^ CD8^+^) and CD4^+^ T cells (CD45^+^ CD3^+^ NK1.1^−^ CD4^+^) and the proportions of Tnaive (CD44^−^ CD62L^+^), Teff (CD44^+^ CD62L^−^), and Tcm (CD44^+^ CD62L^+^) of the total cell population are shown. Percentages of these subsets are shown in **Supplementary Figure 13**. **(C–E)** The absolute numbers of NK cells (CD45^+^ CD3^−^ NK1.1^+^), NKT cells (CD45^+^ CD3^+^ NK1.1^+^), and B cells (CD45^+^ CD3^−^ NK1.1^−^ B220^+^) were calculated. Compilation of two experiments. Each symbol represents data of an individual mouse. n=6 for CON CCR2 WT, n=6 for CON CCR2 KO, n=11 for UT CCR2 WT, n=11 for UT CCR2 KO, n=8 for ART+CQ CCR2 WT, n=10 for ART+CQ CCR2 KO.

**Figure 9:**
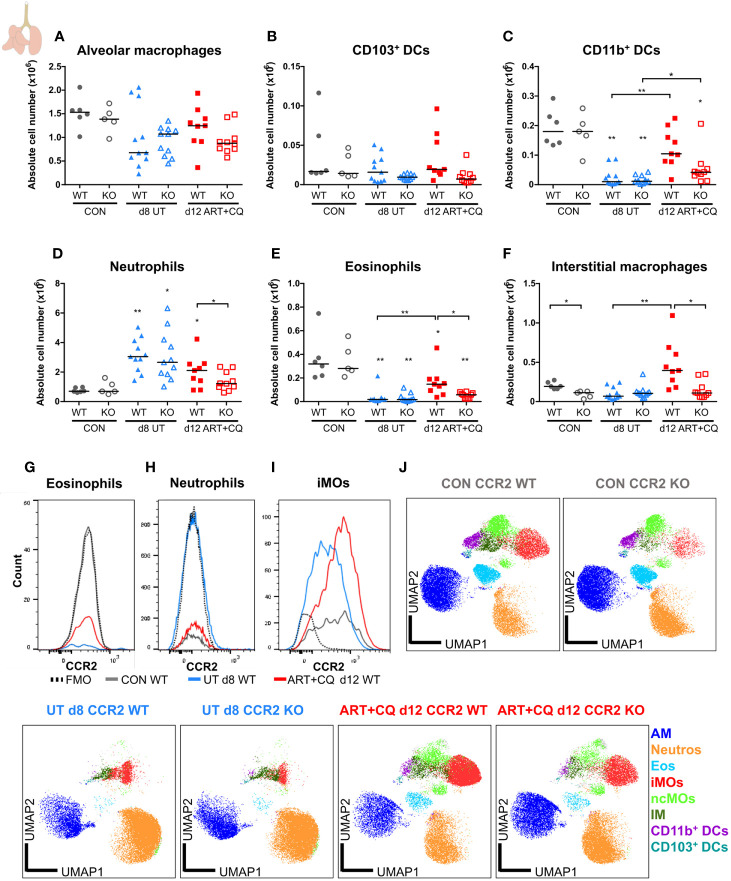
CCR2 is crucial for the return to homeostasis for the myeloid cell populations in the lungs. CCR2 WT and CCR2 KO C57BL/6 mice were infected with *Pb*NK65. Mice were injected daily from 8 until 12 days p.i. with 10 mg/kg ART + 30 mg/kg CQ. Mice were dissected at the indicated days p.i. Leukocytes were isolated from the lungs according to protocol 2 and flow cytometry was performed. **(A–F)** The absolute numbers of alveolar macrophages (AM; CD45^+^ SiglecF^+^ CD11b^int^ CD11c^+^), CD103^+^ dendritic cells (CD103^+^ DCs; CD45^+^ Lin^−^ SiglecF^−^ MHC-II^+^ CD11c^+^ CD103^+^ CD11b^−^), CD11b^+^ dendritic cells (CD11b^+^ DCs; CD45^+^ Lin^−^ SiglecF^−^ MHC-II^+^ CD11c^+^ CD103^−^ CD11b^+^ CD24^+^ CD64^−^), neutrophils (Neutros; CD45^+^ Lin^−^ CD11b^+^ Ly6G^+^), eosinophils (Eos; CD45^+^ CD11b^+^ SiglecF^+^ CD11c^−^), and interstitial macrophages (IM; CD45^+^ Lin^−^ CD11b^hi^ MHC-II^+^ CD64^+^ CD24^−^) in the lungs were calculated. Compilation of two experiments. Each symbol represents data of an individual mouse. n=6 for CON CCR2 WT, n=5 for CON CCR2 KO, n=11 for UT CCR2 WT, n=11 for UT CCR2 KO, n=9 for ART+CQ CCR2 WT, n=10 for ART+CQ CCR2 KO. **(G, H)** Representative flow cytometry plots showing the CCR2 expression of eosinophils **(G)**, neutrophils **(H)**, and iMOs **(I)** in the lungs of CCR2 WT mice at the different time-points. **(J)** Clustering of 24,000 cells combined from two representative samples per condition for the CON CCR2 WT and CON CCR2 KO and from three representative samples per condition for the others. The plots show a two-dimensional representation (UMAP) of the protein expression. Clusters are colored by cell class as defined in panels **(A–F)** and **Figures 7A, B**.

## Results

### Antimalarial Treatment Induces Resolution of Experimental MA-ARDS

C57BL/6 mice were infected with *Pb*NK65, which resulted in the development of MA-ARDS (**Figure 1**). The first clinical disease symptoms appeared 8 days p.i. (**Figure 1C**). MA-ARDS is characterized by the development of alveolar edema, which was measured by an increased protein concentration in the BALF in the untreated, *Pb*NK65-infected (UT) group compared to the uninfected controls (CON) on day 0 p.i. (**Figure 1E**) (31).

Artesunate (ART) is an antimalarial drug that efficiently and rapidly kills the parasites (5). To optimize the antimalarial treatment in our mouse model, two dosages of 40 mg/kg and 10 mg/kg ART (ART 40 and ART 10) were tested. At 8 days p.i., when the first disease symptoms appeared in the *Pb*NK65-infected C57BL/6 mice, antimalarial treatment was started. Both dosages of ART significantly decreased parasitemias, clinical scores, and the levels of alveolar edema, resulting in survival rates of 80–100% from the otherwise lethal MA-ARDS complication (**Supplementary Figure 1**). A combination of 10 mg/kg of ART with 3 mg/kg of dexamethasone (DEX), a synthetic glucocorticoid, was also tested. This combination did not have a beneficial effect compared to ART alone, as parasites were cleared at a slower rate and the level of alveolar edema at 4 days post-treatment was not significantly decreased compared to the untreated group at 1 day post-treatment (**Supplementary Figure 1**). The efficiency of ART 10 was similar compared to ART 40. To limit the anti-inflammatory effects of ART, ART 10 was chosen as the preferred antimalarial treatment dosage. Besides clearing parasitemia, this treatment also resulted in a lower pulmonary mRNA expression of the inflammatory chemokines CCL2 and CXCL10, and of the cytokines tumor necrosis factor-α (TNF-α), IFN-γ, and IL-10 (**Supplementary Figure 2**).

Since recrudescences of parasitemia after the end of treatment with both ART dosages were often observed, for all subsequent experiments, treatment with ART 10 was combined with 30 mg/kg of chloroquine, a slower but longer-acting antimalarial drug (5). Starting this artesunate + chloroquine (ART+CQ) treatment for 5 days when the first disease symptoms appeared (8 days p.i.), resulted in a 60% decrease in parasitemia after one day of treatment (at day 9 p.i) and complete parasite clearance by the end of treatment (at day 12 p.i.) (**Figures 1A, B**). Upon antimalarial treatment, on average 80% of the mice were rescued from the otherwise lethal MA-ARDS complication (**Supplementary Figure 1B**). The clinical score still worsened from 8 to 9 days p.i., but started to decrease at 10 days p.i. until the mice appeared completely healthy again at 12 days p.i. (**Figure 1C**). The body weight recovered from 11 days p.i. onward (**Figure 1D**). An increase in the protein concentration in the BALF was observed at 8 and 9 days p.i. compared to the controls, showing that massive alveolar edema was present (**Figure 1E**). Thereafter, in the mice treated with ART+CQ, alveolar edema decreased and reached control levels at 15 days. Macroscopically, the development of the lung pathology and microhemorrhages were visible by the darkening of the lungs, which is due to microhemorrhages (31) (**Figure 1F**). These signs of pathology also resolved upon ART+CQ treatment.

### Dynamics of Pulmonary Leukocyte Numbers and Activation During the Resolution of MA-ARDS

To determine the dynamics of leukocyte subpopulations in the lungs during the resolution of MA-ARDS, leukocytes were isolated from the lungs and analyzed by multicolor flow cytometry. CD8^+^ T cells are known to be pathogenic in MA-ARDS (7, 9, 31) and their number was significantly increased in the lungs of *Pb*NK65-infected C57BL/6 mice at 8 and 9 days p.i. (**Figure 2A**). Interestingly, the absolute number of CD8^+^ T cells further increased at 12 days p.i. compared to 8 days. At 15 days p.i., their numbers were still higher compared to controls. In contrast, no significant differences in the number of CD4^+^ T cells were observed (**Figure 2B**). The activation phenotype of T cells in the lungs was investigated at 0, 8, 9, 12, and 15 days p.i. Both CD8^+^ and CD4^+^ T cells were activated during infection, resulting in an increased proportion of effector T (Teff) cells and a decreased proportion of naive T (Tnaive) cells (**Figures 2A, B**; **Supplementary Figure 3**). At 12 and 15 days p.i., the Teff cells remained the largest T cell subset in the lungs. No differences were found in the absolute number of NK cells (**Figure 2C**), whereas NKT cell counts were significantly increased after ART+CQ treatment (**Figure 2D**). Transient increases in B cell numbers were observed in ART+CQ-treated mice at 9 and 12 days p.i. compared to the uninfected controls (**Figure 2E**).

In these experiments, no differences in the number of alveolar macrophages (AM) were observed in the lungs (**Figure 3A**). The number of neutrophils increased in the lungs at 9 days p.i., irrespectively of the antimalarial treatment, and remained so at 12 days p.i. (**Figure 3B**). The Ly6C^+^ inflammatory monocytes (iMOs) were increased upon infection and remained increased until 12 days p.i. (**Figure 3C**). The numbers of Ly6C^−^ non-classical monocytes (ncMOs) and dendritic cells (DCs) were increased during resolution (**Figures 3D–E**). Eosinophils largely disappeared from the lungs upon infection and gradually reappeared during resolution (**Figure 3F**). To analyze and depict the myeloid cell populations of the lungs in a more unbiased and gating-independent manner, UMAP plots were generated with the flow cytometry data (**Figure 3G**). The Ly6C^+^ iMOs, Ly6C^−^ ncMOs, and DCs clustered closely together, while the AM, neutrophil, and eosinophil clusters were more distinct. Moreover, clustering alterations were observed for the Ly6C^+^ iMOs and AM upon infection and resolution. This was attributed to differences in the expression of specific markers, in particular CD64/FcγRI, which was increased at 8 days p.i. and decreased during resolution (**Supplementary Figure 4**). This reflects the activation of the monocytes during infection, which is also observed in humans (34, 35).

Overall, these data show that the first phase of resolution (at 12 days p.i.) was not characterized by a decrease of the infiltrating leukocytes. In fact, several leukocyte populations continued to increase during the first days of resolution, when both the alveolar edema and cytokines expression were already decreasing. At 15 days p.i., most leukocyte subpopulations were decreasing and approached control levels.

### Dynamics of Splenic Leukocyte Numbers and Activation in the Resolution of MA-ARDS

The number of CD8^+^ T cells, CD4^+^ T cells, NK cells, and NKT cells were decreased in the spleens at 8 days p.i. compared to the uninfected controls (**Figures 4A–D**). During resolution, the absolute numbers of these cell types gradually increased. B cell numbers were slightly, but significantly downregulated after antimalarial treatment at 9 days p.i. compared to the untreated group at 8 days p.i. (**Figure 4E**). Similarly as in the lungs, the CD8^+^ and CD4^+^ T cells became activated in the spleen upon infection, as was shown by an increased proportion of Teff cells and a decreased proportion of Tnaive cells compared to the uninfected controls, without any change during resolution (**Figures 4A, B**; **Supplementary Figure 5**).

Most myeloid cell populations, such as DCs, eosinophils, iMOs, ncMOs, and red pulp macrophages, were also significantly depleted in the spleen upon infection and gradually increased again during resolution (**Figure 5**). The iMOs and ncMOs already reappeared at 12 days p.i., while it took until 15 days p.i. for the DCs, eosinophils, and red pulp macrophages to return. Splenic neutrophils were not depleted by the infection and gradually increased during resolution (**Figure 5**).

Altogether, in this mouse model, we were able to rescue >80% of the mice from the otherwise lethal MA-ARDS complication, by starting antimalarial treatment (ART+CQ) on the day that the first clinical signs of disease appeared (at 8 days p.i.). The mice completely recovered and the lung inflammation fully resolved upon antimalarial treatment, as was shown by alveolar edema quantification, cytokine and chemokine expression and infiltrating cell determination. These results showed that this mouse model was useful to study the mechanisms and leukocyte involvement in the resolution of inflammation and pathology in MA-ARDS.

### CCR2 Gene Knock-Out Has No Effect on the Development Nor the Resolution of MA-ARDS

Since monocytes and macrophages play important roles in the resolution of many inflammatory pathologies (12), their role was investigated with CCR2 KO mice. We carefully checked the CCR2 KO status at the protein and DNA levels (**Supplementary Figure 6**), and also confirmed that the genetic background of both CCR2 WT and KO mice was >99.95% C57BL/6 (**Supplementary Table 3**).

Parasitemia, clinical score, and loss of body weight were similar in CCR2 KO mice compared to CCR2 WT mice, both during infection and during/after antimalarial treatment (**Figures 6A–C**). Lung pathology, i.e., the amount of alveolar edema and the weight of the left lung, was similarly increased in both the WT and KO mice at 8 days p.i. (**Figures 6D–E**). Moreover, at 9 days p.i., both in the UT as in the ART+CQ group, no difference in lung pathology was observed between the CCR2 KO and CCR2 WT mice (**Supplementary Figure 7**). During resolution, alveolar edema was cleared from the lungs in both the CCR2 KO and CCR2 WT mice, while the weight of the left lung was still increased (**Figures 6D–E**). Also macroscopically, no differences in lung pathology were observed between CCR2 WT and KO mice (**Figure 6F**). In conclusion, CCR2 is neither required for the development of MA-ARDS, nor for the resolution of the pathology upon antimalarial treatment.

### Effect of CCR2 Gene Knock-Out on the Dynamics of Pulmonary and Splenic Leukocyte Populations

Compared to the experiments above (protocol 1), a different isolation protocol (protocol 2) for the pulmonary leukocytes was further used. This adapted protocol allowed for the quantitative detection of DCs, IMs, and a more detailed appreciation of the dynamics of ncMOs, since it resulted in a higher number of total isolated leukocytes without affecting the overall dynamics of pulmonary leukocytes during MA-ARDS development and resolution.

The number of pulmonary iMOs was significantly lower at each time point in the CCR2 KO mice compared to the WT mice (**Figure 7A** and **Supplementary Figure 8**). Except for day 12 p.i., the numbers of ncMO were not affected by the CCR2 KO (**Figure 7B** and **Supplementary Figure 8**). At 12 days p.i., both iMOs and ncMOs were strikingly increased in the lungs of *Pb*NK65-infected WT mice, while this was largely abolished in the infected CCR2 KO group (**Figures 7A, B**). In the spleens of uninfected controls, a significant lower number of iMOs was found in the CCR2 KO compared to the WT mice, whereas no difference was observed for the ncMOs (**Figures 7C, D** and **Supplementary Figure 8**). The number of iMOs and ncMOs in the spleen similarly decreased in CCR2 KO and WT mice upon infection (8 and 9 days p.i.). The reappearance of both the iMOs and ncMOs in the spleen during resolution at 12 days p.i. was also similar in both the KO and WT mice, indicating that this is a CCR2-independent process.

In the spleens, except for the iMOs, none of the studied leukocyte populations showed significant differences between the CCR2 KO and WT mice at any time-point (**Supplementary Figures 9**-**12**).

In the lungs, no significant differences between the CCR2 KO and CCR2 WT mice were found, for any lymphocyte population at any of the analyzed time-points (**Figure 8**; **Supplementary Figures 13** and **14**). In addition, the CCR2 KO had no effect on the number of AM (**Figure 9A** and **Supplementary Figure 15A**). The CD103^+^ and CD11b^+^ DCs disappeared upon infection and reappeared during resolution, although it took until 9 days p.i. for the CD103^+^ DCs to decrease, whereas the CD11b^+^ DCs were already decreased at 8 days p.i. (**Figures 9B, C** and **Supplementary Figures 15B, C**). In CCR2 KO mice, a trend toward reduced numbers of CD103^+^ and CD11b^+^ DCs was observed compared to WT mice during resolution (at 12 days p.i.; **Figures 9B, C**; p = 0.0684 and p = 0.0532, respectively). Upon infection, the neutrophil numbers increased at 8 and 9 days p.i. in both the WT and KO group, whereas at 12 days p.i., less neutrophils were present in the lungs of the CCR2 KO mice compared to the WT mice (**Figure 9D** and **Supplementary Figure 15D**). The eosinophils disappeared from the lungs upon infection and reappeared during resolution in the CCR2 WT mice (**Figure 9E** and **Supplementary Figure 15E**). Interestingly, their reappearance did not occur as efficiently in the CCR2 KO mice. The CCR2 KO thus had an effect on the presence of neutrophils and eosinophils in the lungs during resolution, despite the fact that the neutrophils and eosinophils do not express CCR2 in CCR2 WT mice (**Figures 9G, H**), in contrast to the Ly6C^+^ iMOs (**Figure 9I**). This suggested that the mitigation of eosinophil reappearance during resolution was an indirect effect of the CCR2 KO, probably caused by the decreased numbers of monocytes present in the lungs. Interstitial macrophages decreased at 9 days p.i. and increased at 12 days p.i., but no increase occurred at 12 days p.i. in the CCR2 KO mice (**Figure 9F** and **Supplementary Figure 15F**). Also in the uninfected controls, reduced numbers of interstitial macrophages were observed in the lungs of CCR2 KO mice compared to WT mice. UMAP plots were generated to visualize these flow cytometry data (**Figure 9J**). Like before, the monocyte populations, the DC populations and the interstitial macrophages clustered closely, whereas the neutrophils, eosinophils, and AM clustered more separately. Also, shifts in the clusters between different time-points were observed for the AM and iMOs, and this was attributed to a change in expression of activation markers, in particular CD64. The expression of CD64 was increased on pulmonary iMOs and AM in both the WT and KO mice at 8 days p.i., and decreased again during resolution (**Supplementary Figure 16**).

**Figure 10:**
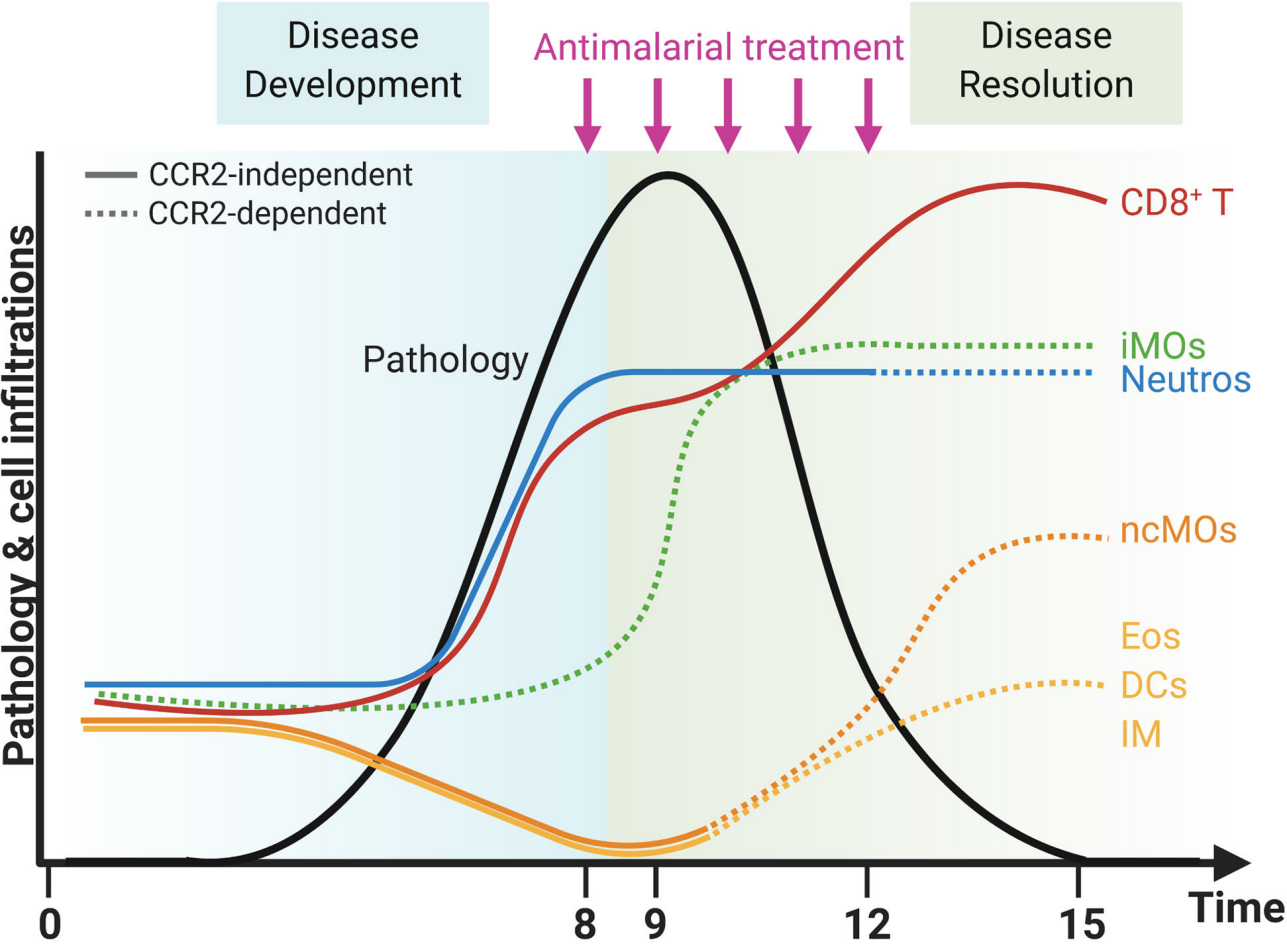
CCR2-dependent dynamics of pulmonary leukocytes during inflammation and resolution. Upon infection with *Pb*NK65 parasites, C57BL/6 mice develop malaria-associated acute respiratory distress syndrome (MA-ARDS). This development of pathology is accompanied by an increase in the number of neutrophils (Neutros) and pathogenic CD8^+^ T cells and a decrease in the number of eosinophils (Eos), dendritic cells (DCs), interstitial macrophages (IM), and non-classical Ly6C^−^ monocytes (ncMOs) in the lungs. Late in the development of MA-ARDS, the number of pulmonary inflammatory Ly6C^+^ monocytes (iMOs) increased as well. Upon antimalarial treatment, mice recover from MA-ARDS with a decrease in clinical symptoms and lung pathology. During resolution, the number of CD8^+^ T cells further increased in the lungs, whereas the number of Neutros and iMOs remained high. In addition, the Eos, DCs, and IM reappeared in the lungs and the ncMOs increased even higher than in control mice during resolution. CCR2 gene KO inhibited several of these dynamic changes, and CCR2-dependent effects were indicated with dashed lines. The number of iMOs present in the lungs was decreased at each time-point in the CCR2 KO mice. In addition, the Neutros remained high during resolution in CCR2 WT mice, but decreased in the CCR2 KO mice. The Eos, DCs, IM, and ncMOs did not reappear or increase during resolution in the lungs of CCR2 KO mice. Created with BioRender.com.

In summary, the lower numbers of Ly6C^+^ iMOs in the lungs in CCR2 KO mice did not affect the development and the resolution of the lung pathology of MA-ARDS. In contrast, the CCR2 gene deletion had an effect on the return to leukocyte homeostasis in the lungs, as was shown by the mitigation of the reappearance of eosinophils and the lack of increase in interstitial macrophages in the CCR2 KO mice compared to the WT mice. In addition, the decrease in neutrophil numbers was more pronounced in the lungs of CCR2 KO mice compared to the WT mice. The overall changes in the composition of pulmonary leukocytes during inflammation and resolution in our MA-ARDS model and the effects of the CCR2 KO on these dynamics are summarized in **Figure 10**.

## Discussion

Pro-resolving therapies have a broad mechanism of action involving modulation of the immune response and stimulation of repair. This is in sharp contrast with anti-inflammatory treatments that merely induce a strong inhibition of the immune response (11, 12, 14). Therefore, pro-resolving therapies are considered to be more efficient than anti-inflammatory drugs to treat severe inflammatory pathologies. In this study, we developed a mouse model to study the resolution of MA-ARDS upon parasite killing. In this model, 80% of the *Pb*NK65-infected mice were rescued from the otherwise lethal MA-ARDS complication when ART+CQ was started on the day that the first disease symptoms appeared. In these mice, parasites were cleared and alveolar edema was resolved. This model allows us to study the molecular mechanisms and leukocyte populations involved in this recovery process, possibly offering a great asset for exploring novel therapies for malaria complications.

Previously, it was shown that 80 mg/kg of DEX, a potent glucocorticoid, resulted in increased survival and decreased lung pathology in *Pb*NK65-infected C57BL/6 (31). In the current study, we tested the effect of DEX on the resolution of MA-ARDS at a dosage of 3 mg/kg, in analogy with the maximum dosage for patients. The use of DEX in combination with ART was not beneficial in inducing resolution, as parasitemia and alveolar edema were cleared less efficiently. This is in line with the previous observation that DEX treatment increased parasitemia, which may explain the detrimental effect when combined with antimalarial treatment (31).

MA-ARDS is a Th1-mediated pathology in which CD8^+^ T cells are pathogenic. Depletion of these cells resulted in the absence of pathology (7, 9, 31). In general, resolution mechanisms of such Th1-mediated pathologies remain poorly understood. Therefore, we performed detailed flow cytometry analyses to characterize the dynamics of leukocyte populations and their activation in lungs and spleen. During infection, both CD4^+^ and CD8^+^ T cells became activated, as was shown by a downregulation of CD62L expression and an upregulation of CD44 expression, which corresponded with a Teff phenotype. In fact, CD8^+^ T cell numbers increased in the lungs during infection and further increased during resolution, while remaining activated. This was also observed by Claser et al. (7). They also demonstrated that IFN-γ is crucial to promote parasite antigen presentation in a MHC-I context by the endothelial cells. CD8^+^ T cells are also a main source of IFN-γ in the lungs (8). Interestingly, we observed that although the number of CD8^+^ T cells further increased during resolution, the expression of IFN-γ at 12 days p.i. was already decreased. Reduced parasite antigen presentation by the endothelial cells in the absence of IFN-γ may thus explain why lung pathology is already decreased at 12 days p.i.

In the lungs, Ly6C^−^ ncMOs decreased during infection but increased during resolution, while Ly6C^+^ iMOs were upregulated both upon infection and during resolution. Moreover, CD64, an activation marker of monocytes, was increased on pulmonary Ly6C^+^ iMOs and alveolar macrophages after infection. Increased CD64 expression on monocytes was also observed in patients with malaria, systemic lupus erythematosus (SLE) and sepsis (34–37). In malaria patients, CD64 upregulation was observed on circulating classical, intermediate and non-classical monocytes of malaria patients (34). CD64 expression correlated with a more severe pathology, such as decreased renal function and high CRP levels, in SLE patients (36). Interestingly, in severe septic patients, the expression on monocytes was higher in patients who survived, compared to patients who died (35, 37). During resolution of MA-ARDS in our model, CD64 expression started decreasing at 12 days p.i., and was fully downregulated to control levels at 15 days p.i. Therefore, similarly to lupus, CD64 expression also parallels the inflammation in MA-ARDS and its subsequent resolution (36).

Our data indicate that CCR2^+^ Ly6C^+^ iMOs are neither involved in the development nor the resolution of MA-ARDS. In the absence of CCR2, monocytes are retained in the bone marrow during homeostatic conditions and cannot migrate to the site of inflammation during infection or inflammation (25). In other studies on the effect of the CCR2 KO on the development of MA-ARDS or malaria-associated acute lung injury, similarly no or only modest differences between CCR2 WT and KO mice are detected (8, 27). Our data correspond largely to the data of Galvao-Filho et al., who also observed that the number of Ly6C^+^ iMOs was lower in the lungs of *Pb*NK65-infected CCR2 KO mice compared to WT mice. Only at 9 days p.i., they observed a reappearance of Ly6C^+^ iMOs in the CCR2 KO mice, which was not observed in our model (8). Our data indicate that during resolution (at 12 days p.i.), the number of Ly6C^+^ iMOs increased in the lungs of both the CCR2 WT and KO mice, although to a much lower extent in the CCR2 KO mice. This may be explained by several processes, such as CCR2-independent egress from the bone marrow, local proliferation, or extramedullary hematopoiesis in the spleen.

Although monocytes and macrophages are important producers of cytokines, we confirm previous studies showing that CCR2 is not crucial in the development of MA-ARDS. Interestingly, Galvao-Filho et al. showed that the pathogenesis is mediated by CCR4-dependent tumor necrosis factor α- and inducible nitric oxide synthase-producing DCs (tip-DCs) (8). Importantly, the absence of a role for CCR2 in the recovery from MA-ARDS is surprising. Monocytes and macrophages are often described as essential players during resolution (13). In fact, several studies have shown that the CCL2/CCR2 axis is crucial for the resolution of various pathologies. In particular, in atherosclerosis and myocardial infarction models, the use of siCCR2 and CCR2 KO mice resulted in a smaller necrotic area and delayed debris clearance with no occurrence of regression (28, 38, 39). The absence of CCR2 also prevented resolution in models of skin wounds, liver fibrosis, and post-operative ileus (29, 40, 41). CCL2 treatment, improved efferocytosis, and subsequent resolution in bacterial pneumonia and increased survival from bleomycin-induced lung injury (42, 43). This effect of CCL2 treatment was, at least in the bleomycin-induced lung injury model, mediated by CCR2, since CCR2 is the main receptor for CCL2 and CCL2 treatment was ineffective in CCR2 KO mice (43). Importantly, resolution of inflammation has been well-characterized in Th2-related diseases, such as helminth infections. In these studies, monocytes and macrophages are described to be crucial because of the switch in macrophage phenotype from the pro-inflammatory “M1” to the reparative “M2-like” phenotype. In contrast, much less is known about the resolution of Th1-related inflammation, in particular in malaria. Our results thus emphasize that different leukocyte populations and processes might be important for inducing resolution of Th1-related diseases, such as malaria, compared to Th2-related diseases. The CCR2 gene deletion resulted not only in reduced numbers of Ly6C^+^ iMOs, but also of Ly6C^−^ ncMOs and interstitial macrophages, and a trend for reduced CD103^+^ and CD11b^+^ DCs during resolution in the lungs. In CCR2 WT mice, a significant proportion of Ly6C^−^ ncMOs, interstitial macrophages and DCs expresses CCR2. Therefore, CCR2 deficiency may have a direct effect on the absolute number of these populations. Alternatively, it may also be an indirect effect caused by the lower numbers of Ly6C^+^ iMOs present in the lungs during resolution, since iMOs are considered precursors for DCs, ncMOs and monocyte-derived macrophages (44–46). Upregulation of Ly6C^+^ iMOs in the circulation and monocyte-derived macrophages in the tissues is observed during inflammatory responses (44). During inflammation, the Ly6C^+^ iMOs infiltrate the inflamed tissues to engulf dying cells and subsequently differentiate to Ly6C^−^ ncMOs, which are involved in tissue repair mechanisms. Ly6C^+^ iMOs are also thought to differentiate into cells resembling Tip-DCs or “M1” macrophages, whereas the Ly6C^−^ ncMOs differentiate into “M2” macrophage-like cells (44–46). In contrast to the effect on interstitial macrophages, the deletion of CCR2 did not affect the number of AM. AM are under steady-state conditions derived from the yolk sac of the embryo or differentiated from fetal liver- or bone marrow-derived monocytes (44, 45). They also have a self-renewing capacity regulated by colony stimulating factor-1 (CSF-1) and granulocyte-macrophage colony stimulating factor (GM-CSF), so they do not necessarily rely on replenishment by iMOs during homeostasis and inflammation. The red pulp macrophages in the spleen also do not rely on CCR2, since they reappeared during resolution in both the CCR2 WT and KO mice.

Interestingly, the reappearance of pulmonary eosinophils, after their decrease during infection, was mitigated in CCR2 KO mice. Since eosinophils do not express CCR2, the indirect effect of the CCR2 KO is likely caused by the diminished number of CCR2^+^ Ly6C^+^ iMOs present in the lungs. Reparative “M2-like” macrophages may provide an ideal environment to attract eosinophils to the lungs, thus the lack of these cells in the CCR2 KO may explain the mitigated reappearance of eosinophils. Eosinophils are described to play a role during resolution by producing specialized pro-resolving lipid mediators, such as protectin D1, and cytokines, IL-4 and IL-13, resulting in the inhibition of neutrophil infiltration and the modulation of the macrophage phenotype (47–49). More specifically, IL-4 and IL-13 induce the “M1” to “M2-like” phenotype switch of macrophages, whereas protectin D1 stimulates macrophage activity to clear the apoptotic neutrophils (47, 50). However, our data suggest that eosinophils are not essential for resolution, since their delayed reappearance in the lungs does not affect the resolution of pathology. In contrast to pulmonary eosinophils, splenic eosinophils did reappear in the spleen during resolution, independently of CCR2. The pulmonary neutrophil numbers were decreased in CCR2 KO mice during resolution. Similarly to the eosinophils, neutrophils did not express CCR2 in CCR2 WT mice, suggesting that the effect observed in the CCR2 KO mice is an indirect consequence.

In conclusion, a new mouse model to study the mechanisms and the involvement of leukocyte populations in the resolution of MA-ARDS was established. This model offers a great asset to elucidate resolution mechanisms and to explore novel therapies for malaria complications, as pro-resolving therapies may be superior over anti-inflammatory therapies. Our data with the CCR2 KO mice indicated that CCR2^+^ Ly6C^+^ iMOs were neither involved in the development nor the resolution of MA-ARDS. In contrast, these cells were important for the leukocytes to return to homeostasis during resolution in CCR2 KO mice. This was indicated by a delayed replenishment of eosinophils and interstitial macrophages.

## Data Availability Statement

The original contributions presented in the study are included in the article/**Supplementary Material**. Further inquiries can be directed to the corresponding author.

## Ethics Statement

The animal study was reviewed and approved by The Animal Ethics Committee of the KU Leuven (License LA1210186, project P049/2018, Belgium).

## Author Contributions

EP, T-TP, LV, QR, HP and SK performed the experiments. EP and QR analyzed the data. PVdS, EP and GO conceived the study. EP and PVdS wrote the first drafts of the manuscript. EP, T-TP, LV, HP, SK, GO and PVdS critically read and edited the manuscript. All authors contributed to the article, read the article and approved the final version.

## Funding

This study was supported by Research Foundation-Flanders (F.W.O.-Vlaanderen, project G086215N and G097318N, G0C9720N) and the Research Fund of the KU Leuven (C1 project C16/17/010). EP is a recipient of the L’Oréal-Unesco Women for Sciences PhD fellowship, T-TP and HP are recipients of an aspirant PhD fellowship of the F.W.O.-Vlaanderen, LV holds a junior Postdoc fellowship of the F.W.O.-Vlaanderen and PVdS is a Research Professor at the KU Leuven.

## Conflict of Interest

The authors declare that the research was conducted in the absence of any commercial or financial relationships that could be construed as a potential conflict of interest.
