# Corrigendum: CCR2 Is Dispensable for Disease Resolution but Required for the Restoration of Leukocyte Homeostasis Upon Experimental Malaria-Associated Acute Respiratory Distress Syndrome

**DOI:** 10.3389/fimmu.2022.926032

**Published:** 2022-06-01

**Authors:** Emilie Pollenus, Thao-Thy Pham, Leen Vandermosten, Queeny Robalo, Hendrik Possemiers, Sofie Knoops, Ghislain Opdenakker, Philippe E. Van den Steen

**Affiliations:** ^1^Laboratory of Immunoparasitology, Department of Microbiology, Immunology and Transplantation, Rega Institute for Medical Research, KU Leuven, University of Leuven, Leuven, Belgium; ^2^Laboratory of Immunobiology, Department of Microbiology, Immunology and Transplantation, Rega Institute for Medical Research, KU Leuven, University of Leuven, Leuven, Belgium

**Keywords:** malaria, inflammation, resolution, monocytes, immunology, parasitology, eosinophils

“Queeny Robalo” was not included as an author in the published article. The corrected Author contributions statement appears below.

## Author Contributions

EP, T-TP, LV, QR, HP and SK performed the experiments. EP and QR analyzed the data. PVdS, EP and GO conceived the study. EP and PVdS wrote the first drafts of the manuscript. EP, T-TP, LV, HP, SK, GO and PVdS critically read and edited the manuscript. All authors contributed to the article, read the article and approved the final version.

Furthermore, one picture of the lungs in **Figure 1F** in the published article was inadvertently duplicated and mislabeled as “ART+CQ d15” instead of “ART+CQ d14”. The corrected **Figure 1** appears below.

**Figure 1:**
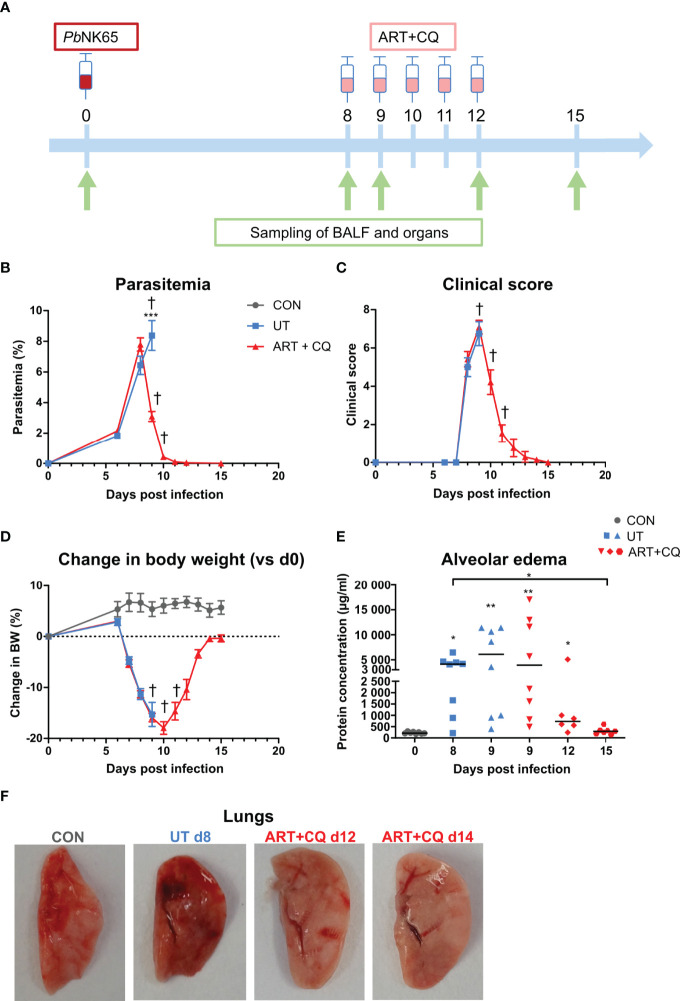
A mouse model to study the resolution of malaria-associated acute respiratory distress syndrome (MA-ARDS) after antimalarial treatment. C57BL/6 mice were infected with PbNK65. Mice were injected daily from 8 until 12 days p.i. with 10 mg/kg ART + 30 mg/kg CQ (ART+CQ). **(A)** Schematic representation of the timing of infection and antimalarial treatments in the mouse model. **(B)** Parasitemia was determined using Giemsa-stained blood smears. **(C)** The clinical score was monitored daily starting at 6 days p.i. **(D)** The change in body weight was calculated compared to day 0 p.i. starting at 6 days p.i. **(B, D)** Compilation of two experiments. Data are means ± SEM. n=8 for uninfected controls (CON), n=8–16 for the infected untreated group (UT), n=7–21 for the infected ART+CQ-treated group. **(E)** The protein concentration in the BALF was determined as a measure of alveolar edema. Compilation of two experiments. Each symbol represents data of an individual mouse. n=8 for CON on day 0, UT at 8 and 9 days p.i and ART+CQ at 9 days p.i., n=6 for ART+CQ at 12 days p.i., n=7 for ART+CQ at 15 days p.i. **(F)** Representative pictures of the left lung.

The authors apologize for these errors and state that this does not change the scientific conclusions of the article in any way. The original article has been updated.

## Publisher’s Note

All claims expressed in this article are solely those of the authors and do not necessarily represent those of their affiliated organizations, or those of the publisher, the editors and the reviewers. Any product that may be evaluated in this article, or claim that may be made by its manufacturer, is not guaranteed or endorsed by the publisher.

