# Supplementary material for: CCR2 Is Dispensable for Disease Resolution but Required for the Restoration of Leukocyte Homeostasis Upon Experimental Malaria-Associated Acute Respiratory Distress Syndrome
Source: Front Immunol. 2021 Feb 16;11:628643. doi: 10.3389/fimmu.2020.628643 (PMC7921736; doi:10.3389/fimmu.2020.628643)
Supplement: Supplementary file 1 [file DataSheet_1.pdf]

Supplementary methods: gating strategies

Lung and spleen cells were isolated and stained for flow cytometry. After exclusion of red blood cells, debris and doublets of cells, all live cells (LD^-) were gated. The leukocytes were identified by CD45 staining. For the lymphoid panel on the lungs, the autofluorescent alveolar macrophages were gated out by plotting LD versus FSC-A as those cells otherwise result in false positive staining for lymphoid cell markers. For each gating strategy, the gating of an uninfected control and of a *Pb*NK65-infected mice on day 8 p.i. is shown below. LD, Live/dead.

Gating strategy for the lymphoid panel for pulmonary leukocytes

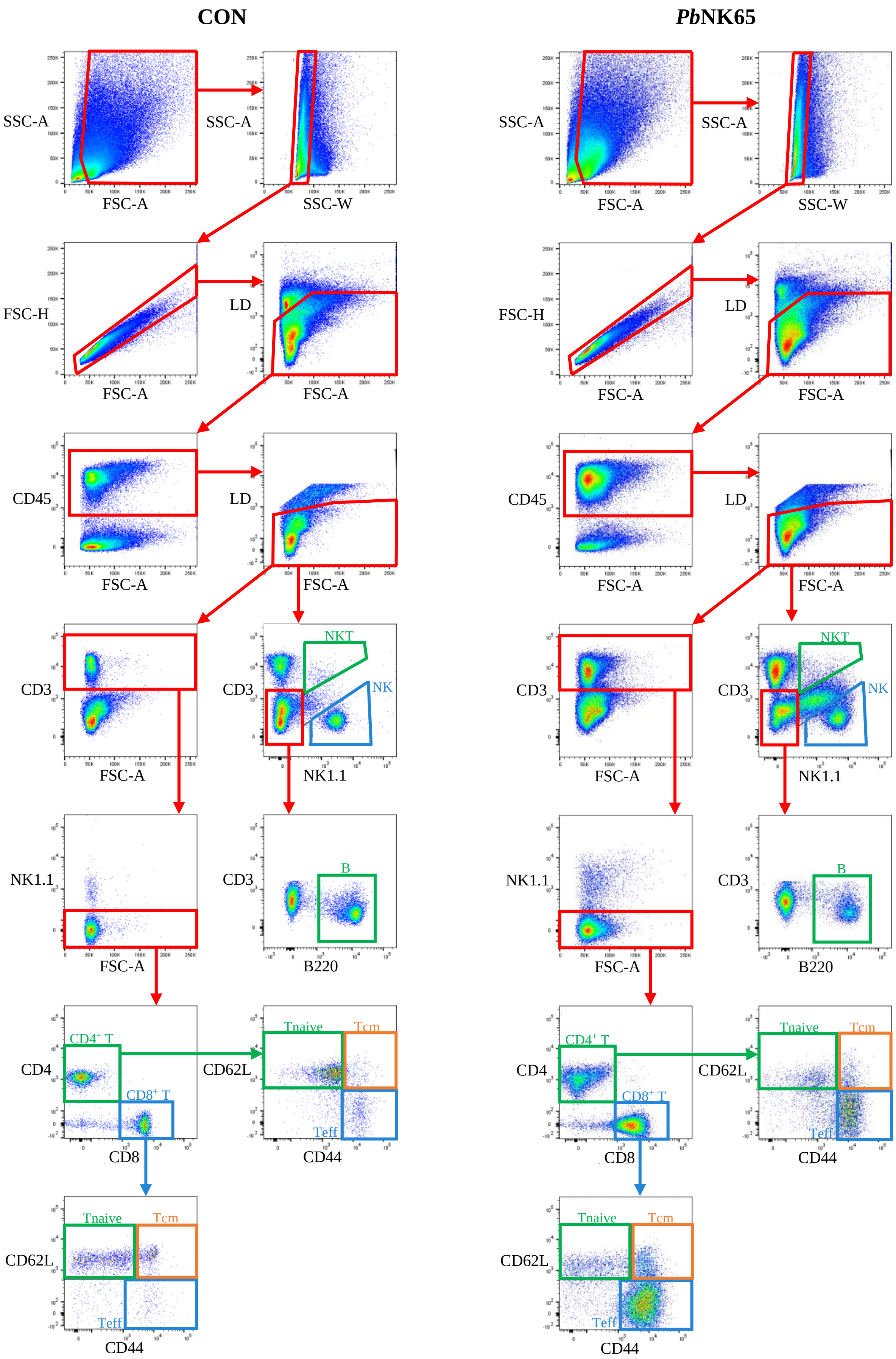

Gating strategy for the lymphoid panel for splenocytes

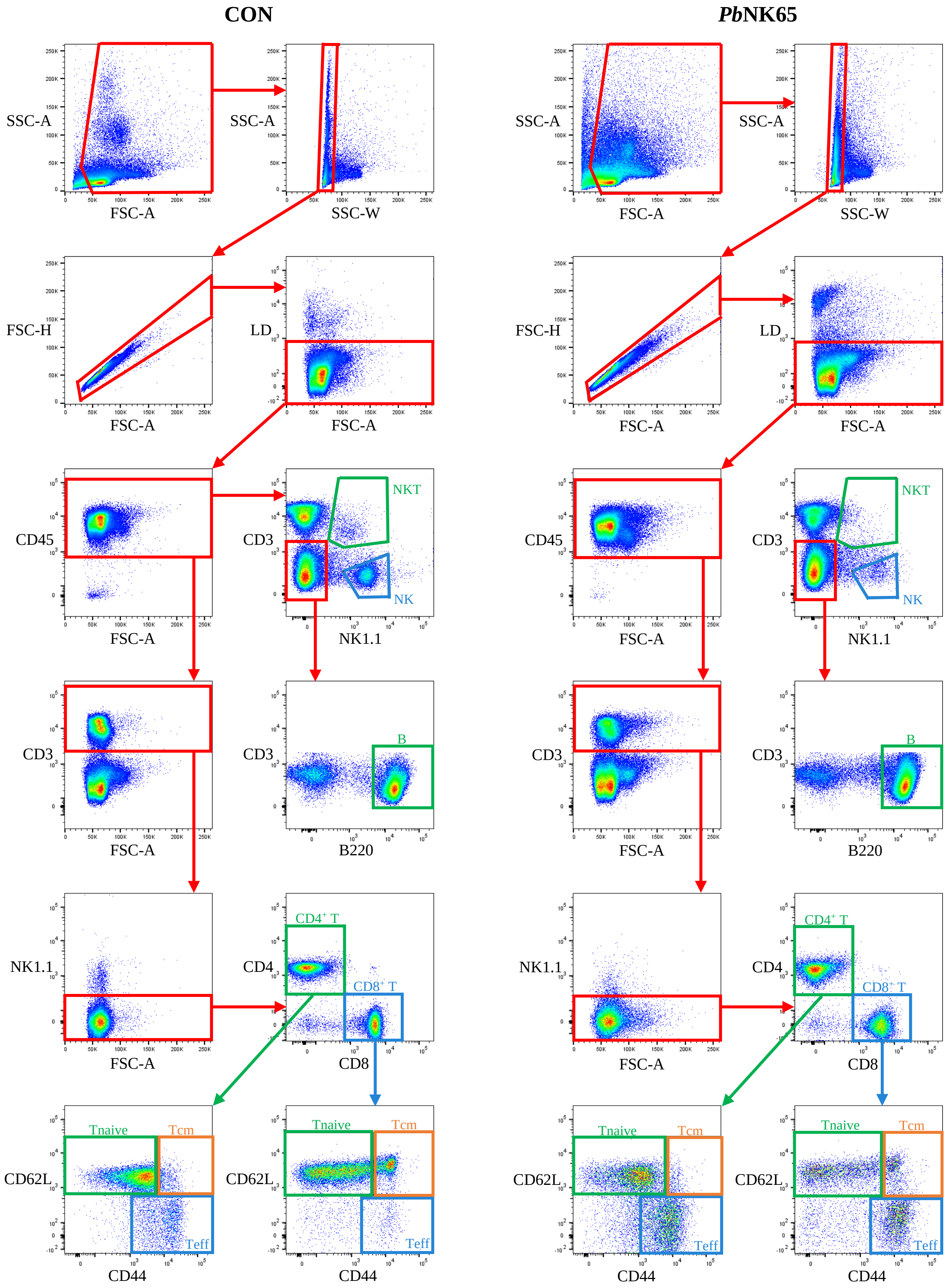

Gating strategy for the myeloid panel for pulmonary leukocytes

CON

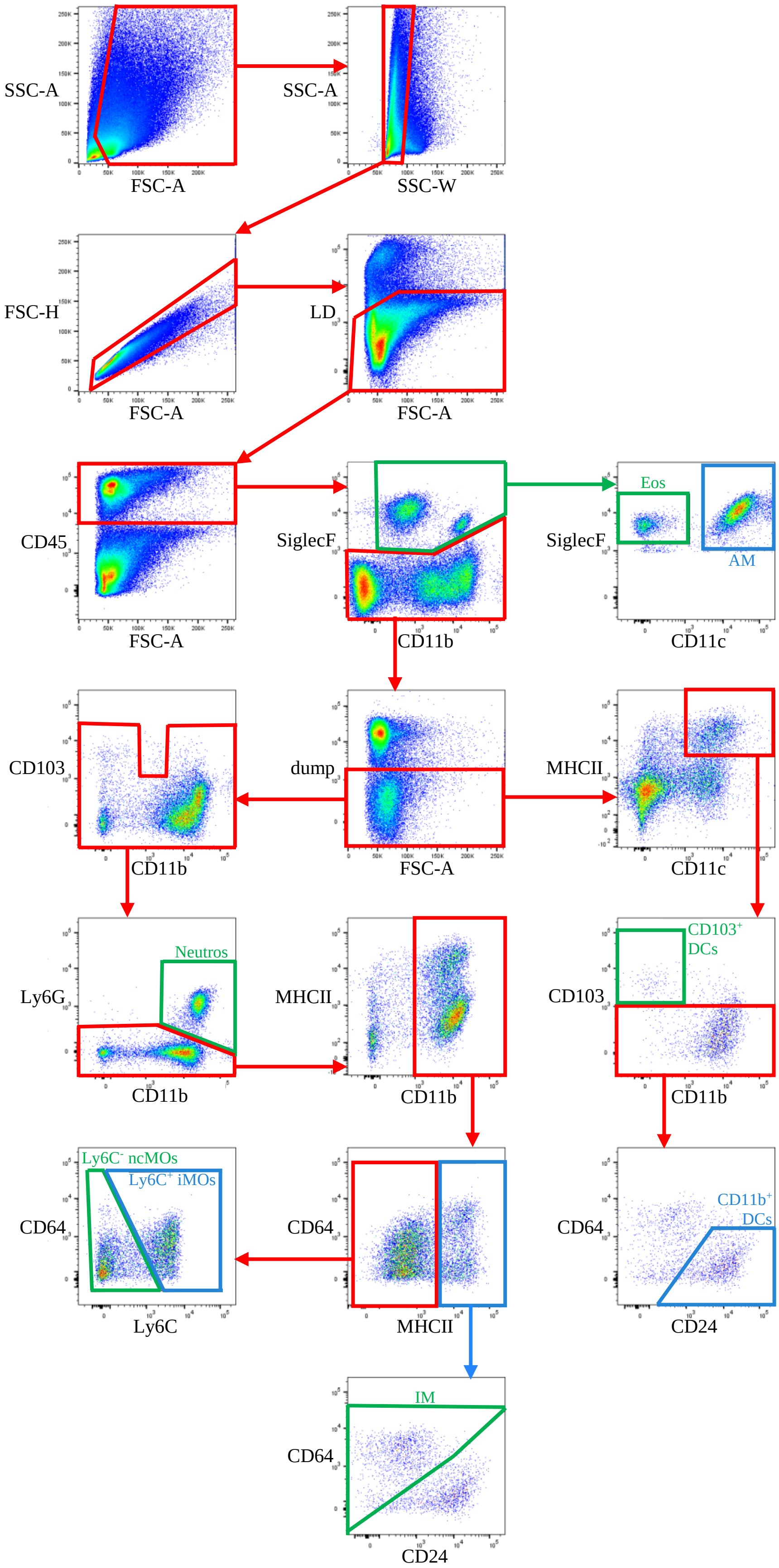

***Pb*NK65**

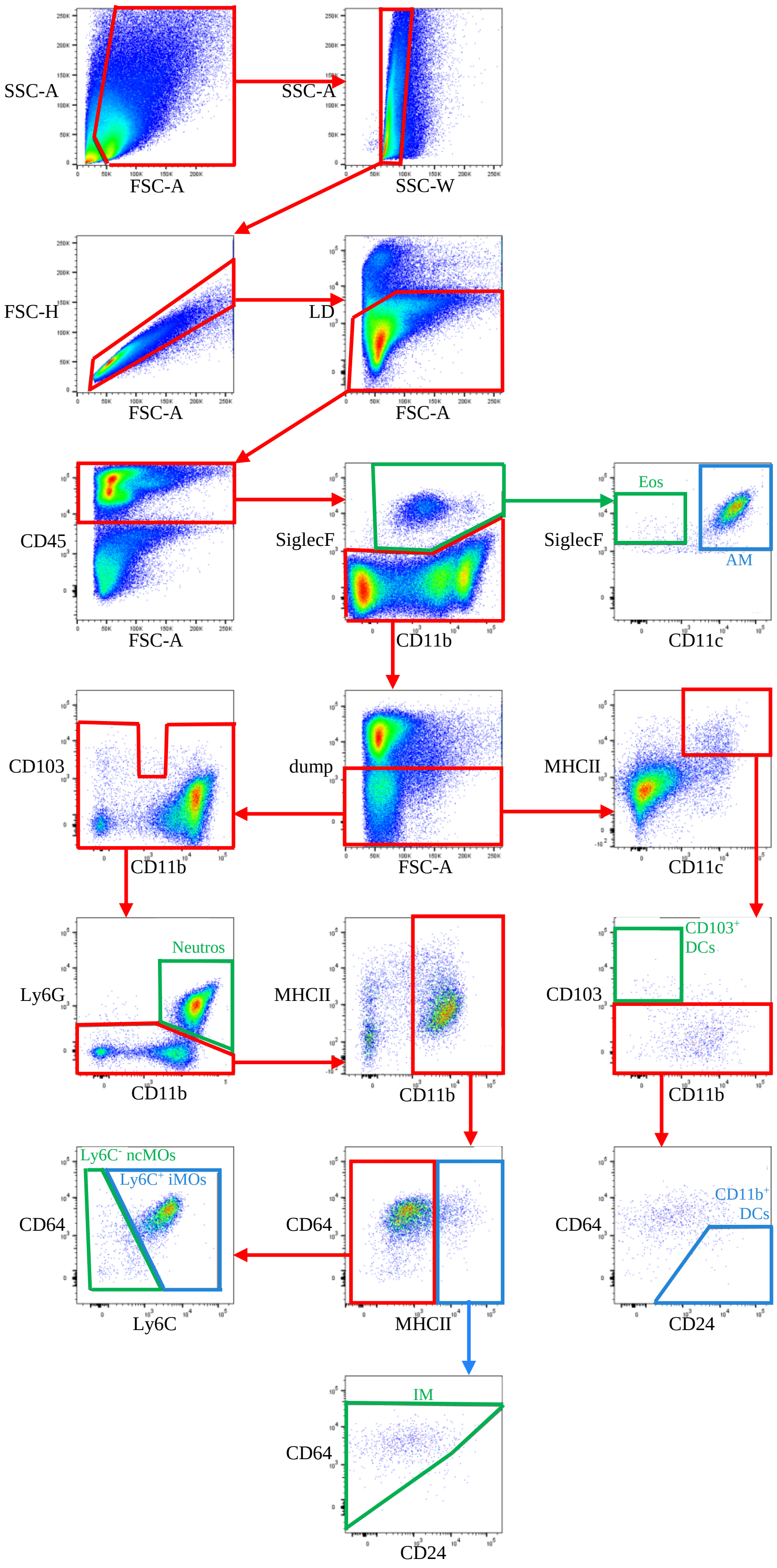
SSC-A
FSC-A
SSC-A
SSC-W
FSC-H
FSC-A
LD
FSC-A
CD45
FSC-A
SiglecF
CD11b
SiglecF
Eos
AM
CD11c
CD103
CD11b
dump
FSC-A
MHCII
CD11c
Ly6G
Neutros
CD11b
MHCII
CD11b
CD103
CD103+ DCs
CD11b
CD64
Ly6C- ncMOs
Ly6C+ iMOs
Ly6C
CD64
MHCII
CD64
CD11b+ DCs
CD24
CD64
IM
CD24

Gating strategy for the myeloid panel for splenocytes

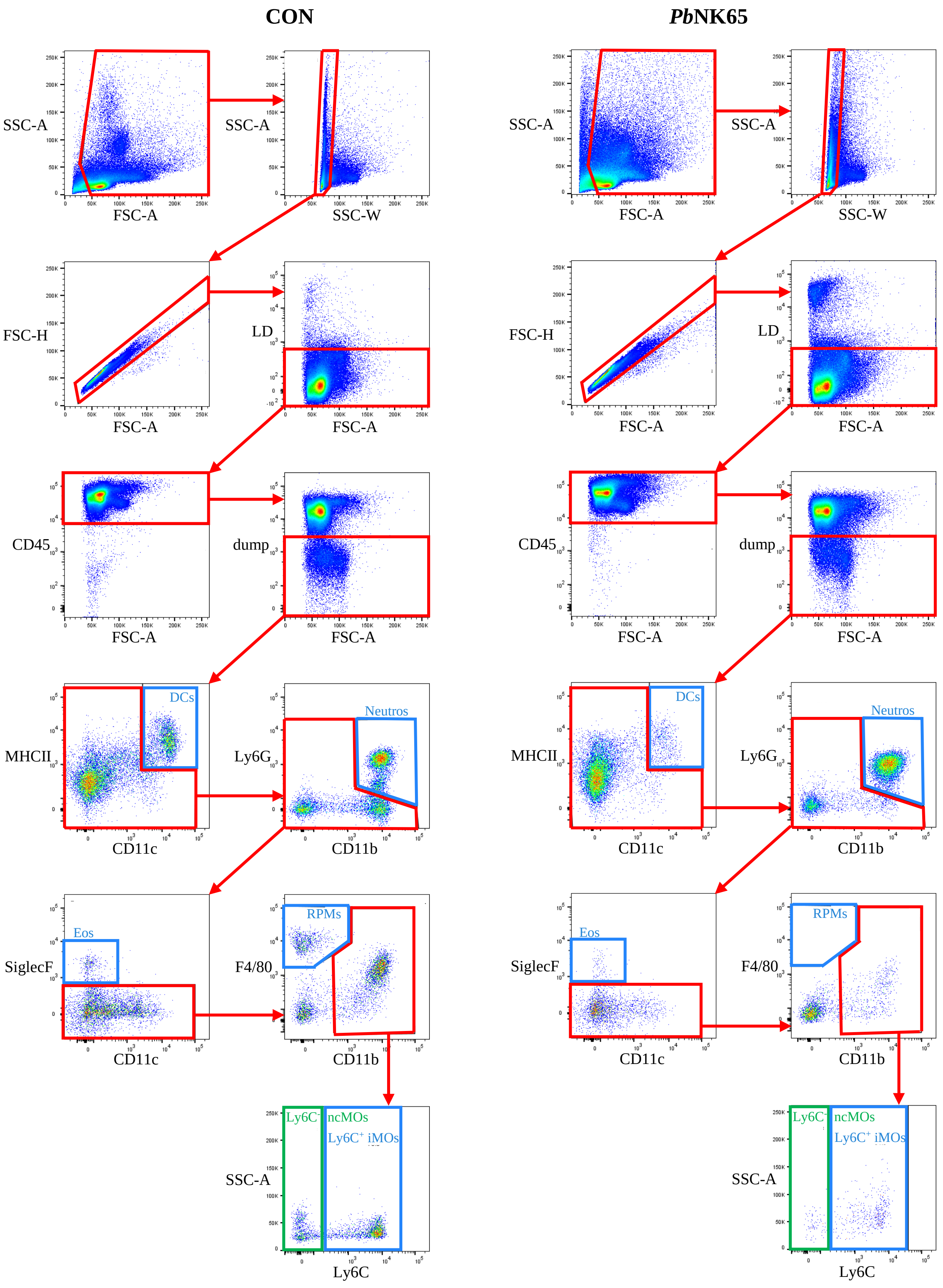

Gating strategy for the CCR2 panel for pulmonary leukocytes

CON

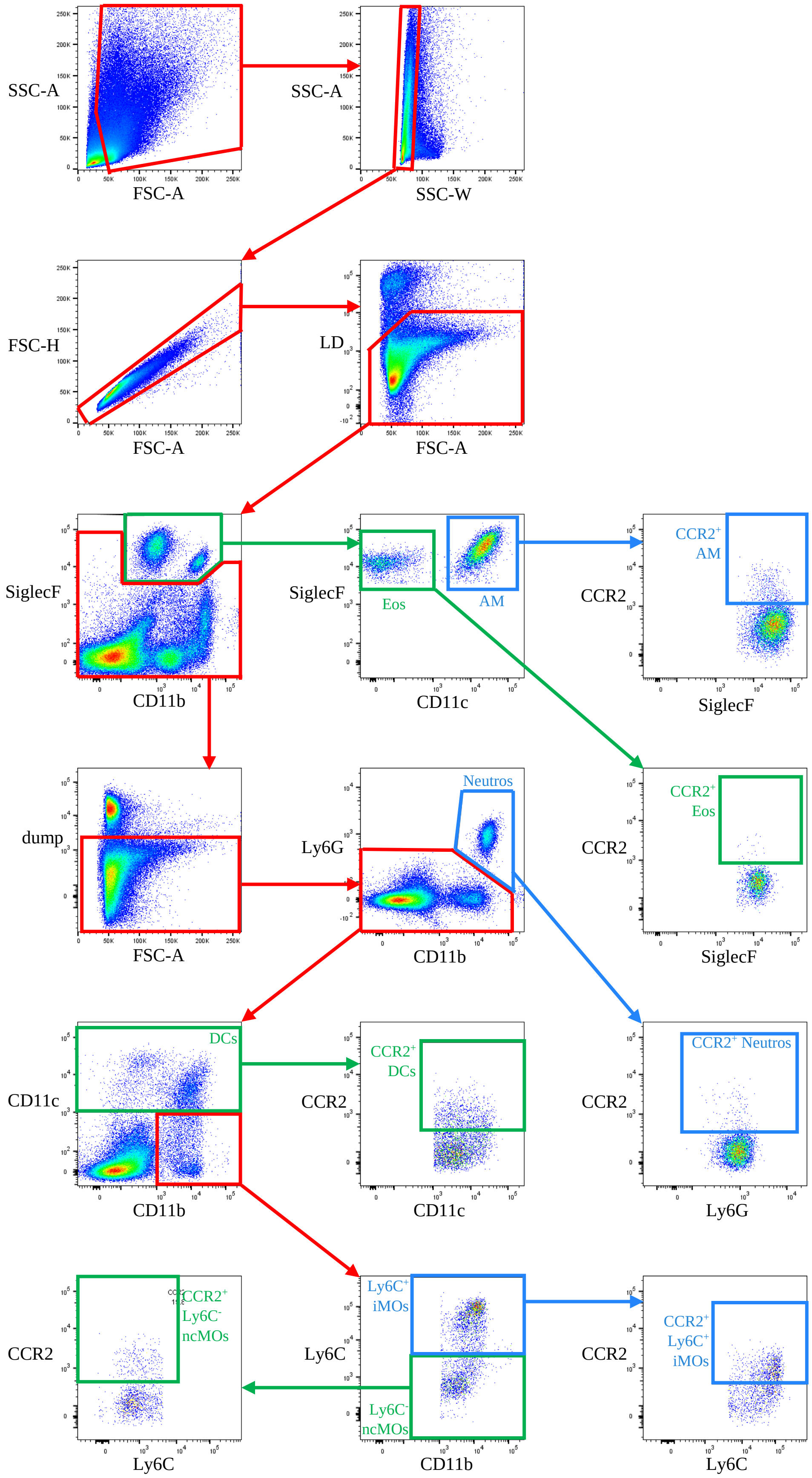

***Pb*NK65**

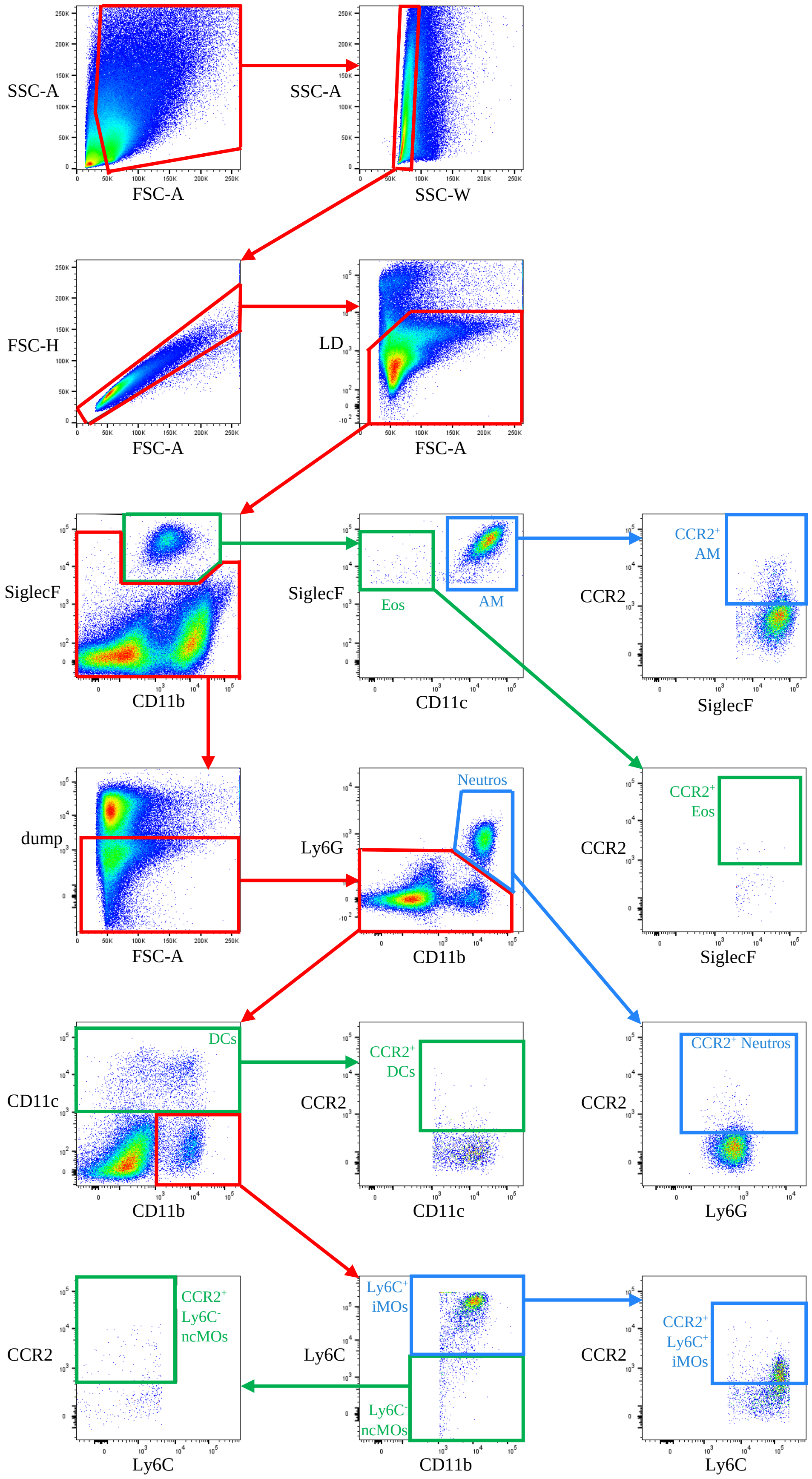
SSC-A
FSC-A
SSC-A
SSC-W
FSC-H
FSC-A
LD
FSC-A
SiglecF
CD11b
SiglecF
CD11c
Eos
AM
CCR2+ AM
CCR2
SiglecF
dump
FSC-A
Ly6G
CD11b
Neutros
CCR2+ Eos
CCR2
SiglecF
CD11c
CD11b
DCs
CCR2+ DCs
CCR2
CD11c
CCR2+ Neutros
CCR2
Ly6G
CCR2
Ly6C
CCR2+ Ly6C- ncMOs
Ly6C+ iMOs
Ly6C- ncMOs
Ly6C
CD11b
CCR2+ Ly6C+ iMOs
CCR2
Ly6C

Gating strategy for the CCR2 panel for splenocytes

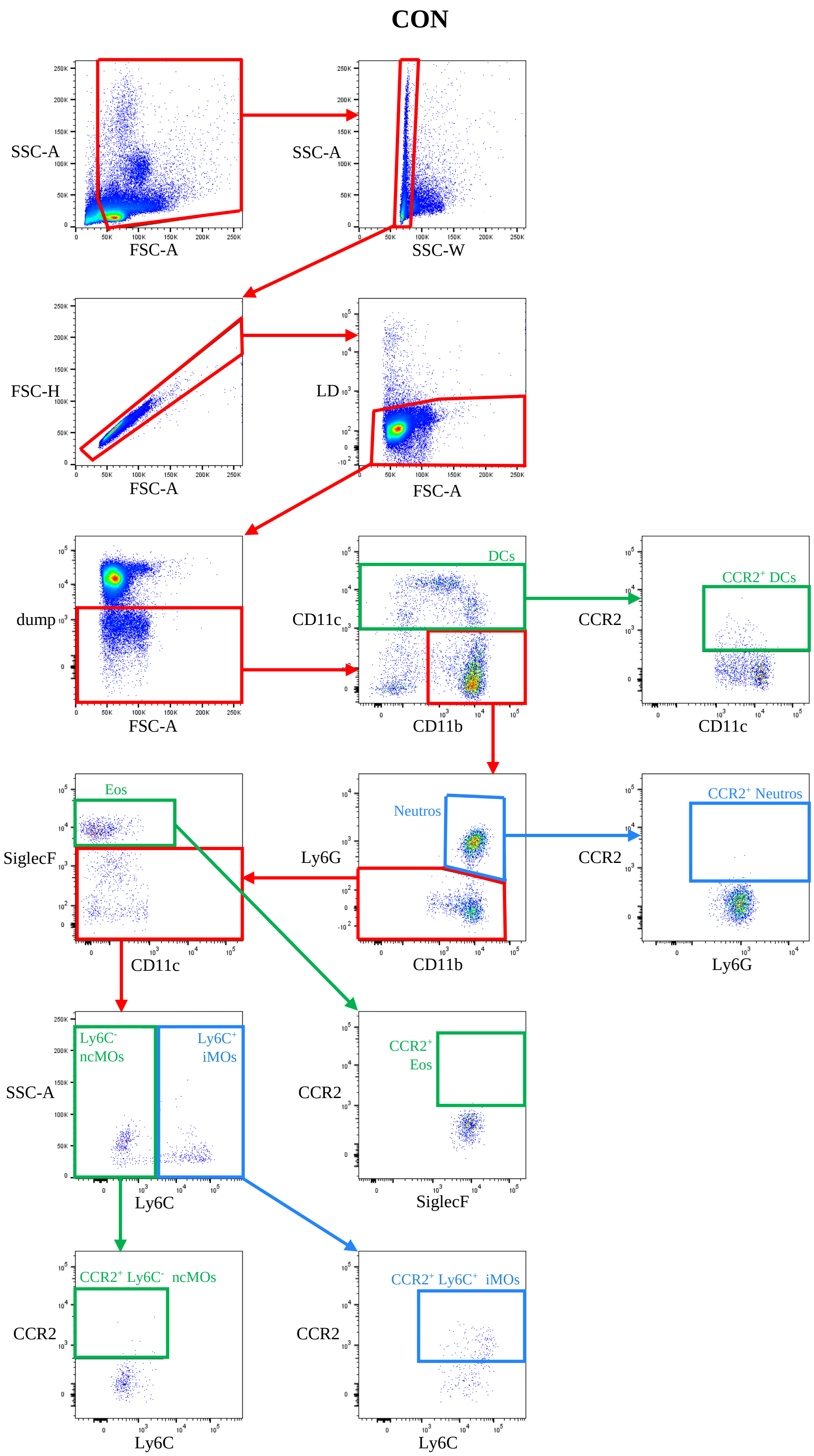

***Pb*NK65**

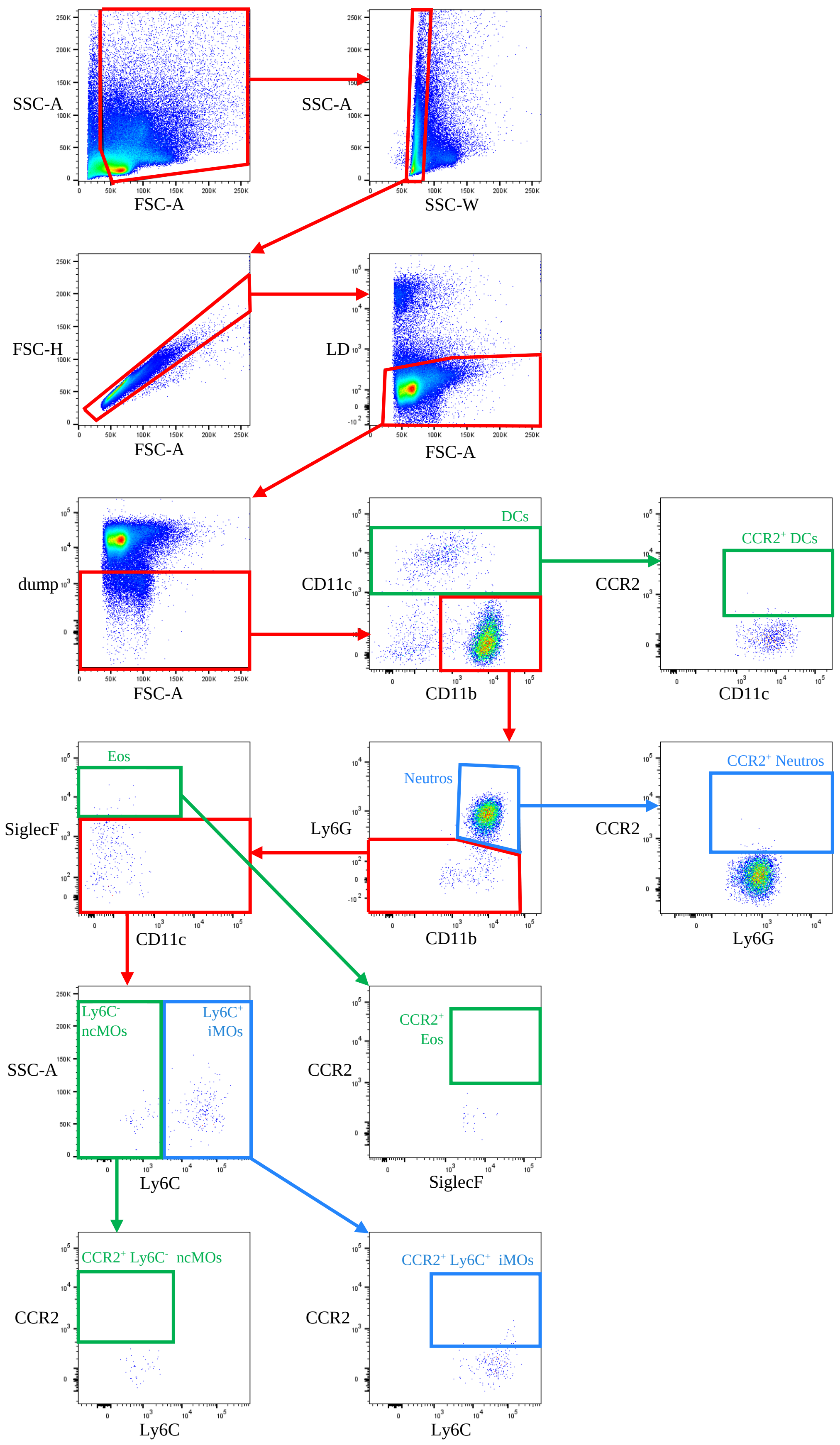
SSC-A
FSC-A
SSC-A
SSC-W
FSC-H
FSC-A
LD
FSC-A
dump
FSC-A
DCs
CD11c
CD11b
CCR2⁺ DCs
CCR2
CD11c
Eos
SiglecF
CD11c
Neutros
Ly6G
CD11b
CCR2⁺ Neutros
CCR2
Ly6G
Ly6C⁻
ncMOs
Ly6C⁺
iMOs
SSC-A
Ly6C
CCR2⁺
Eos
CCR2
SiglecF
CCR2⁺ Ly6C⁻ ncMOs
CCR2
Ly6C
CCR2⁺ Ly6C⁺ iMOs
CCR2
Ly6C
