# Supplementary material for: CCR2 Is Dispensable for Disease Resolution but Required for the Restoration of Leukocyte Homeostasis Upon Experimental Malaria-Associated Acute Respiratory Distress Syndrome
Source: Front Immunol. 2021 Feb 16;11:628643. doi: 10.3389/fimmu.2020.628643 (PMC7921736; doi:10.3389/fimmu.2020.628643)
Supplement: Supplementary file 2 [file DataSheet_2.pdf]

Supplementary Figures

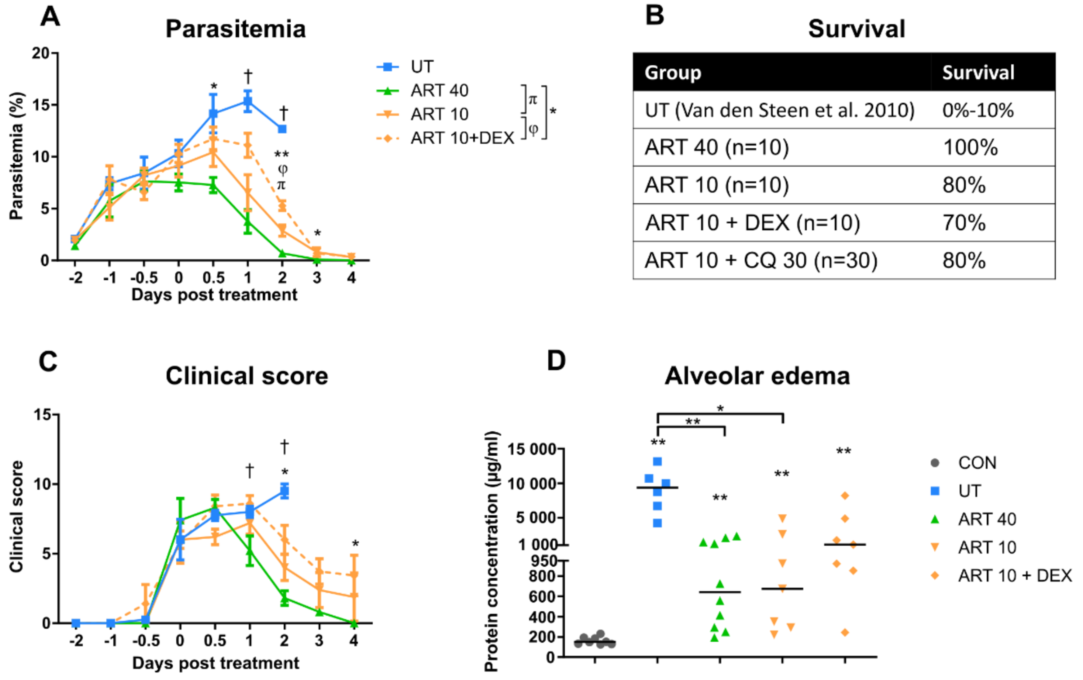

Group	Survival
UT (Van den Steen et al. 2010)	0%-10%
ART 40 (n=10)	100%
ART 10 (n=10)	80%
ART 10 + DEX (n=10)	70%
ART 10 + CQ 30 (n=30)	80%

Supplementary Figure 1. Comparison of different antimalarial treatments in *Pb*NK65-infected C57BL/6 mice.

C57BL/6 mice were infected with *Pb*NK65. Mice were injected daily for four days with 40 mg/kg ART, 10 mg/kg ART or 10 mg/kg ART + 3 mg/kg dexamethasone (DEX) starting when the first disease symptoms appeared. Mice in the untreated (UT) group were injected with carrier-solution (0.4% $NaHCO_3$ in 0.9% NaCl). (A) Parasitemia was determined daily using Giemsa-stained blood smears. Significant differences are indicated according to the symbol code presented in the legend. (B) Proportion of the mice that survived until 4 days p.t. was calculated. n=10 for ART 40, n=10 for ART 10, n=10 for ART 10 + DEX, n= 30 for ART 10 + CQ 30. UT was based on data of previous experiments (31) (C) The clinical score was monitored daily. (A,C) Compilation of two experiments. Data are means ± SEM. n=2-8 for UT, n=5-10 for ART 40, n=5-10 for ART 10, n= 3-10 for ART 10 + DEX. (D) The protein concentration in the BALF was determined as a measure of alveolar edema at 1 day p.t. for the UT group and at 4 days p.t. for the ART 40, ART 10 and ART 10 + DEX groups. Compilation of two experiments. Each symbol represents data of an individual mouse. n = 8 for CON, n = 6 for UT, n=10 for ART 40, n=7 for ART 10, n=7 for ART 10 + DEX.

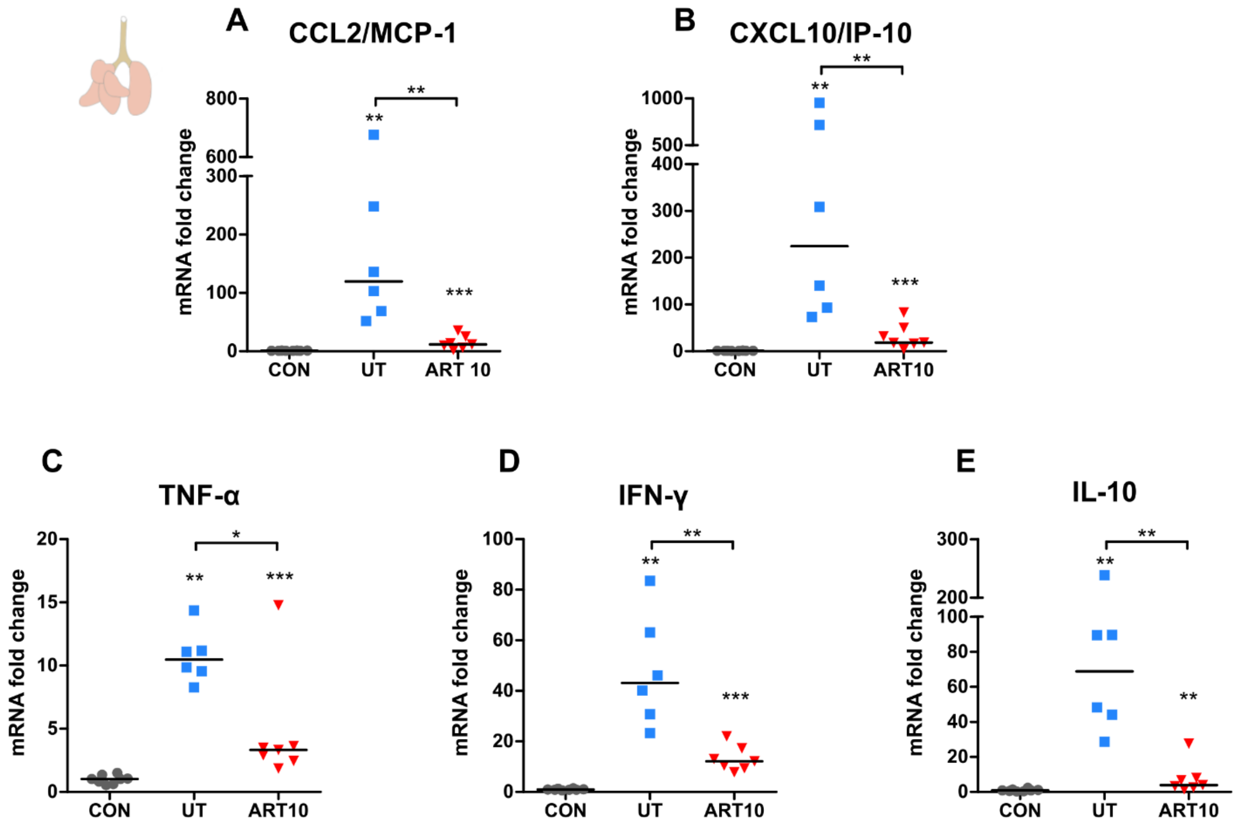

Supplementary Figure 2. Antimalarial treatment reduces the expression of pro-inflammatory chemokines and cytokines.

C57BL/6 mice were infected with *Pb*NK65. Mice were injected daily for four days with 10 mg/kg ART starting when the first disease symptoms appeared. Mice in the untreated (UT) group were injected with carrier-solution (0.4% $NaHCO_3$ in 0.9% NaCl). The difference in the mRNA fold expression in the left lungs on the day of dissection (1 day p.t. for UT group and 4 days p.t. for the ART 10 group) was determined for the indicated molecules. Compilation of two experiments. Each symbol represents data of an individual mouse. n = 8 for CON, n = 6 for UT and n = 7 for ART 10.

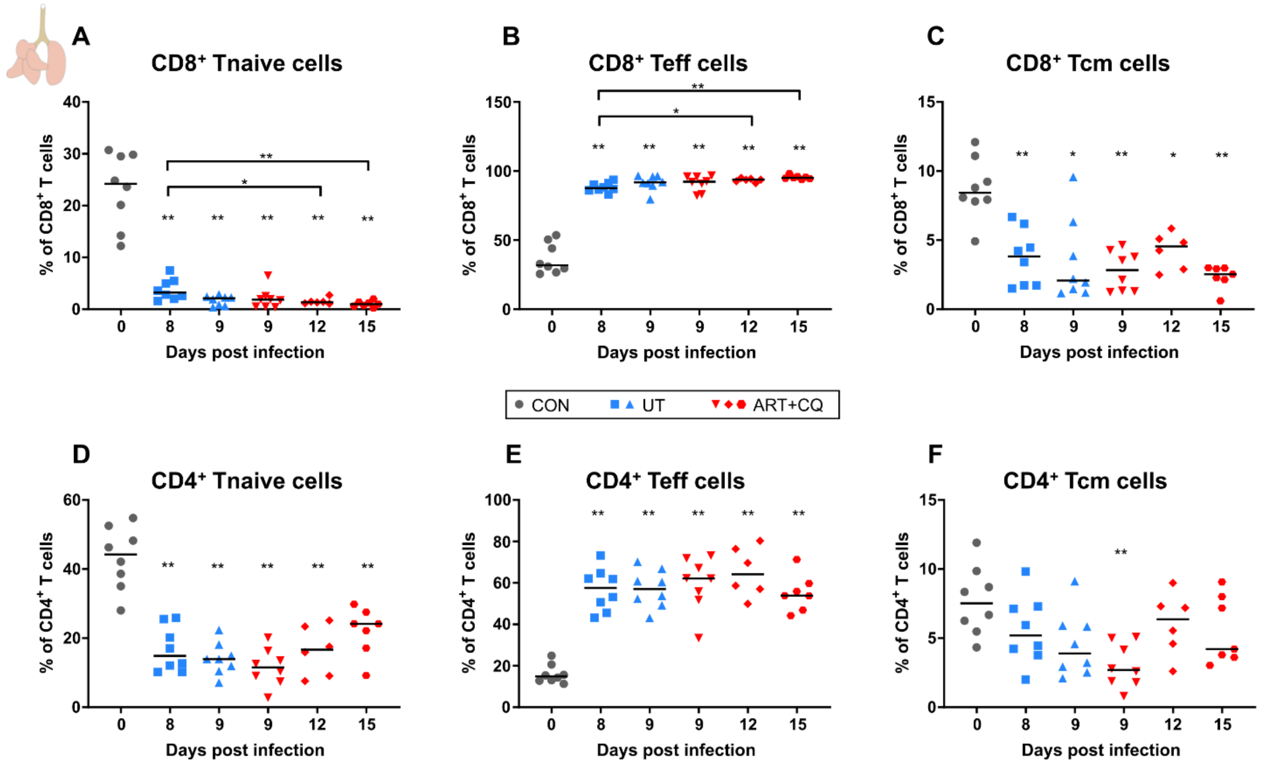

Supplementary Figure 3. Activation status of CD4⁺ and CD8⁺ T cells in the lungs during resolution.

C57BL/6 mice were infected with *Pb*NK65. Mice were injected daily from 8 until 12 days p.i. with 10 mg/kg ART + 30 mg/kg CQ. Mice were dissected at the indicated days p.i. Leukocytes were isolated from the lungs according to protocol 1 and flow cytometry was performed. (A-C) The percentages of Tnaive ($CD44^-$ $CD62L^+$), Teff ($CD44^+$ $CD62L^-$) and Tcm ($CD44^+$ $CD62L^+$) of the total $CD8^+$ T cell population are shown. (D-F) The percentages of Tnaive ($CD44^-$ $CD62L^+$), Teff ($CD44^+$ $CD62L^-$) and Tcm ($CD44^+$ $CD62L^+$) of the total $CD8^+$ T cell population are shown. Compilation of two experiments. Each symbol represents data of an individual mouse. n=8 for CON on day 0, UT at 8 and 9 days p.i and ART+CQ at 9 days p.i., n=6 for ART+CQ at 12 days p.i., n=7 for ART+CQ at 15 days p.i.

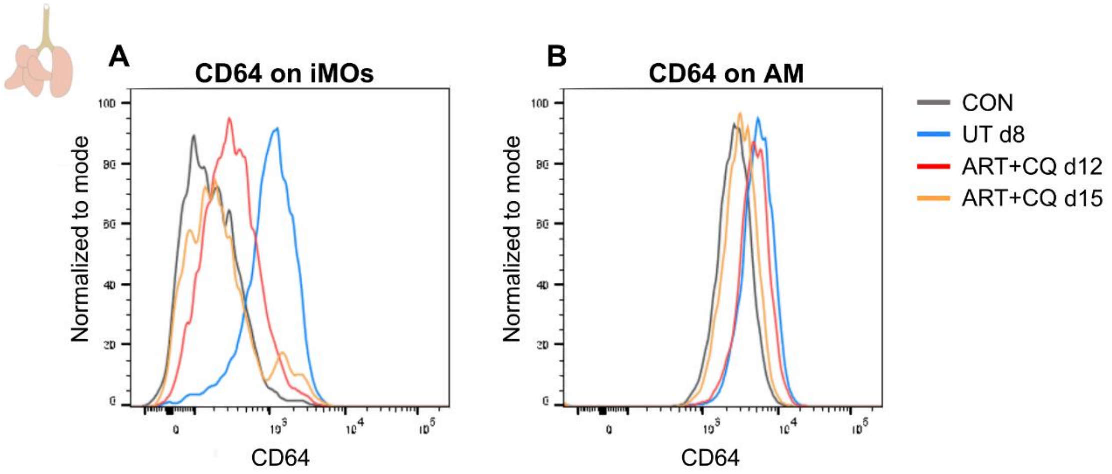

Supplementary Figure 4. Dynamics of CD64 expression by iMOs and AM during resolution.

C57BL/6 mice were infected with *Pb*NK65. Mice were injected daily from 8 until 12 days p.i. with 10 mg/kg ART + 30 mg/kg CQ. Mice were dissected at the indicated days p.i. Leukocytes were isolated from the lungs according to protocol 1 and flow cytometry was performed. Representative histograms of the expression of CD64 on inflammatory monocytes (iMOs, (A); $CD45^+$ Lin^- $CD11b^{hi}$ $MHC\text{-}II^-$ $Ly6C^+$) and alveolar macrophages (AM, (B); $CD45^+$ $SiglecF^+$ $CD11b^{int}$ $CD11c^+$) are shown.

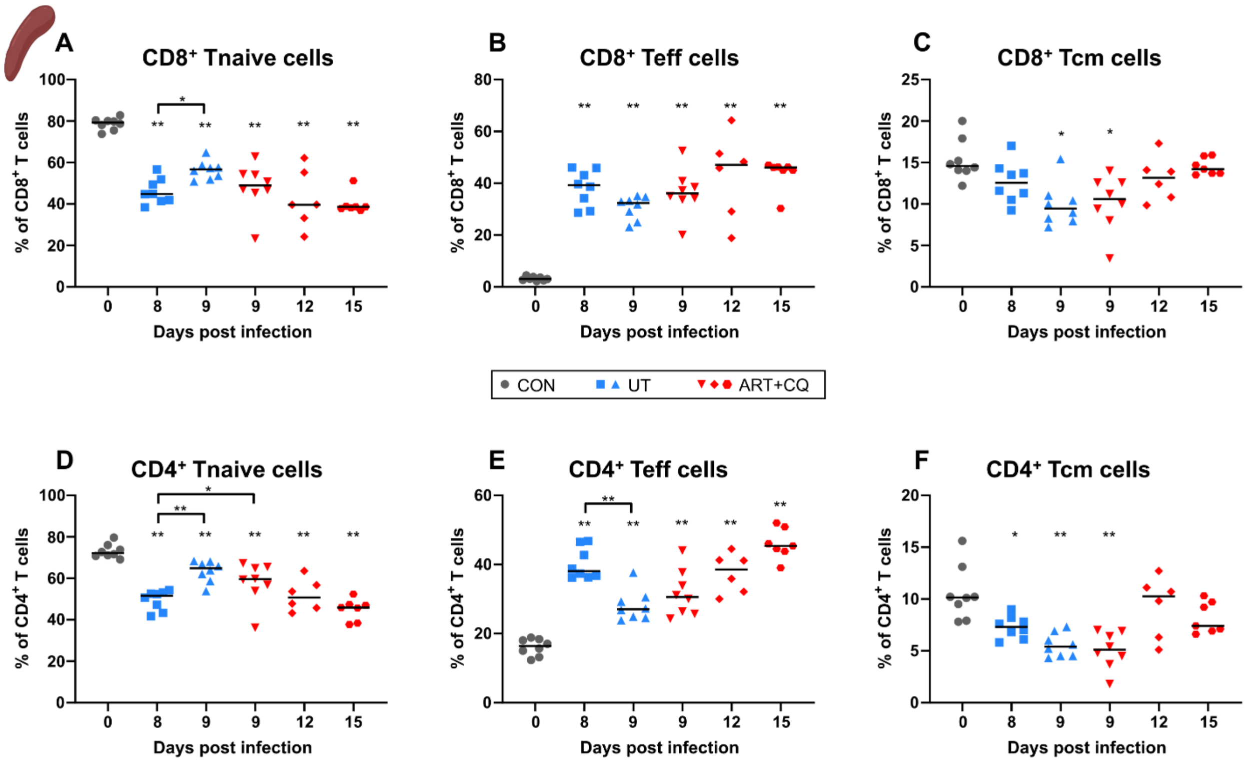

Supplementary Figure 5. Activation status of CD4$^+$ and CD8$^+$ T cells in the spleen during resolution.

C57BL/6 mice were infected with *Pb*NK65. Mice were injected daily from 8 until 12 days p.i. with 10 mg/kg ART + 30 mg/kg CQ. Mice were dissected at the indicated days p.i. Leukocytes were isolated from the spleen and flow cytometry was performed. (A-C) The percentages of Tnaive (CD44$^-$ CD62L$^+$), Teff (CD44$^+$ CD62L$^-$) and Tcm (CD44$^+$ CD62L$^+$) of the total CD8$^+$ T cell population are shown. (D-F) The percentages of Tnaive (CD44$^-$ CD62L$^+$), Teff (CD44$^+$ CD62L$^-$) and Tcm (CD44$^+$ CD62L$^+$) of the total CD4$^+$ T cell population are shown. Compilation of two experiments. Each symbol represents data of an individual mouse. n=8 for CON on day 0, UT at 8 and 9 days p.i and ART+CQ at 9 days p.i., n=6 for ART+CQ at 12 days p.i., n=7 for ART+CQ at 15 days p.i.

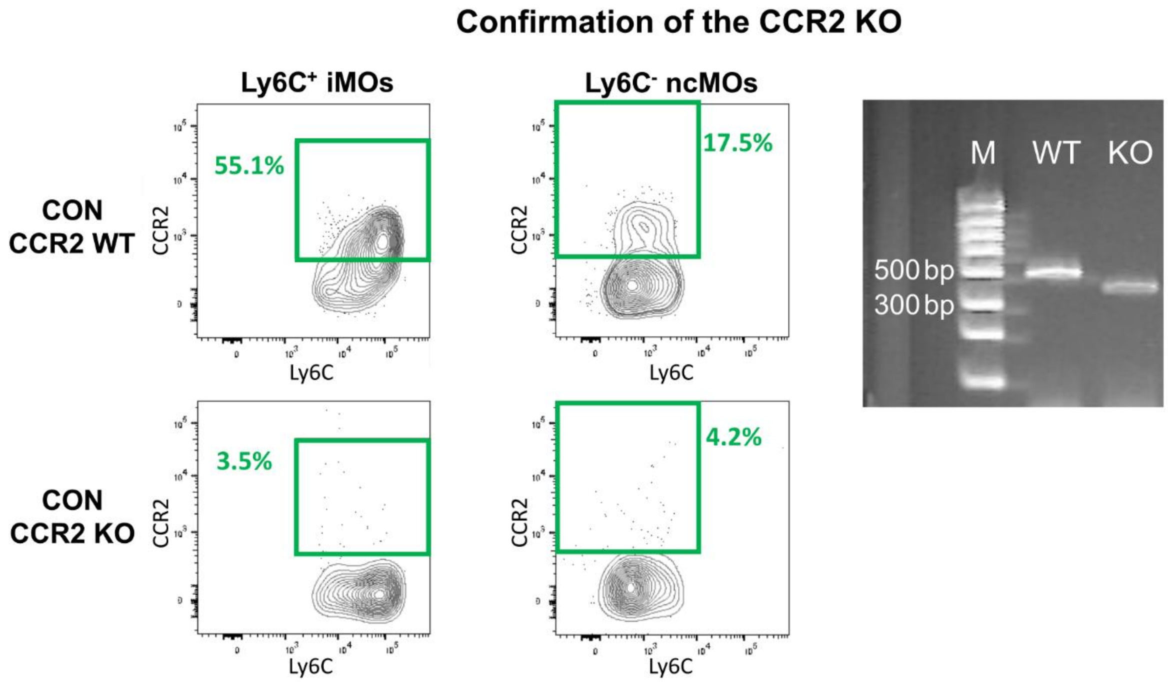

Supplementary Figure 6. Confirmation of the CCR2 knock-out.

Leukocytes were isolated from the lungs according to protocol 2 and flow cytometry was performed. Representative flow cytometry plots showing the CCR2 expression of Ly6C^{+} iMOs (CD45^{+} Lin^{-} CD11b^{hi} MHC-II^{-} Ly6C^{+}) and Ly6C^{-} ncMOs (CD45^{+} Lin^{-} CD11b^{hi} MHC-II^{-} Ly6C^{-}) in the lungs of both uninfected CCR2 WT and KO mice. Confirmation of the CCR2 KO on DNA level using PCR and gel electrophoresis (WT band: 494 bp, KO band: ~390 bp).

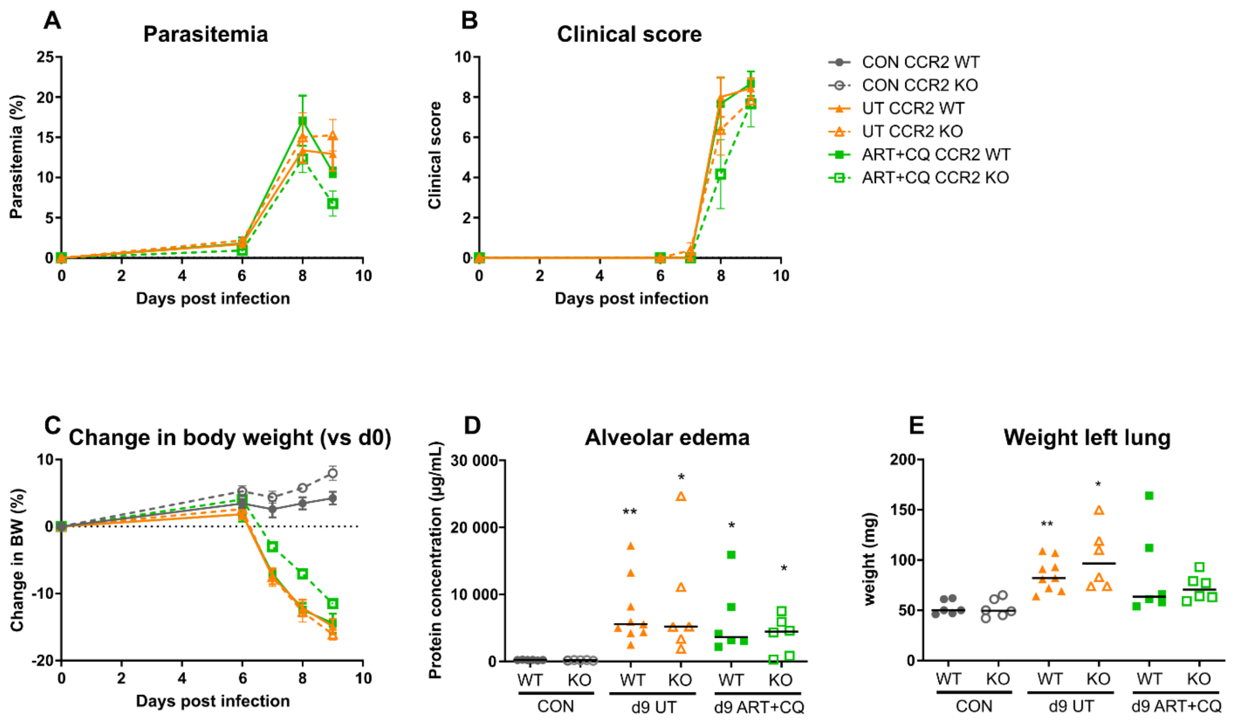

Supplementary Figure 7. CCR2 gene knock-out has no effect on the development nor the resolution of MA-ARDS at 9 days p.i.

CCR2 WT and CCR2 KO C57BL/6 mice were infected with *Pb*NK65. Mice were injected at 8 days p.i. with 10 mg/kg ART + 30 mg/kg CQ. (A) Parasitemia was determined daily using Giemsa-stained blood smears. (B) The clinical score was monitored daily starting at 6 days p.i. (C) The change in body weight was calculated compared to day 0 p.i. starting at 6 days p.i. (A-C) Compilation of two experiments, except ART+ CQ CCR2 WT and KO group only one experiment. Data are means ± SEM. n=6 for CON CCR2 WT, n=6 for CON CCR2 KO, n=9-10 for UT CCR2 WT, n=6-8 for UT CCR2 KO, n=6 for ART+CQ CCR2 WT, n=6 for ART+CQ CCR2 KO. (D-E) Lung pathology was quantified based on the protein concentration in the BALF (D) and the weight of the left lung (E) at 9 days p.i. for the UT group and the ART+CQ group. Compilation of two experiments, except ART+ CQ CCR2 WT and KO group only one experiment. Each symbol represents data of an individual mouse. n=6 for CON CCR2 WT, n=5 for CON CCR2 KO, n=9 for UT CCR2 WT, n=6 for UT CCR2 KO, n=6 for ART+CQ CCR2 WT, n=6 for ART+CQ CCR2 KO.

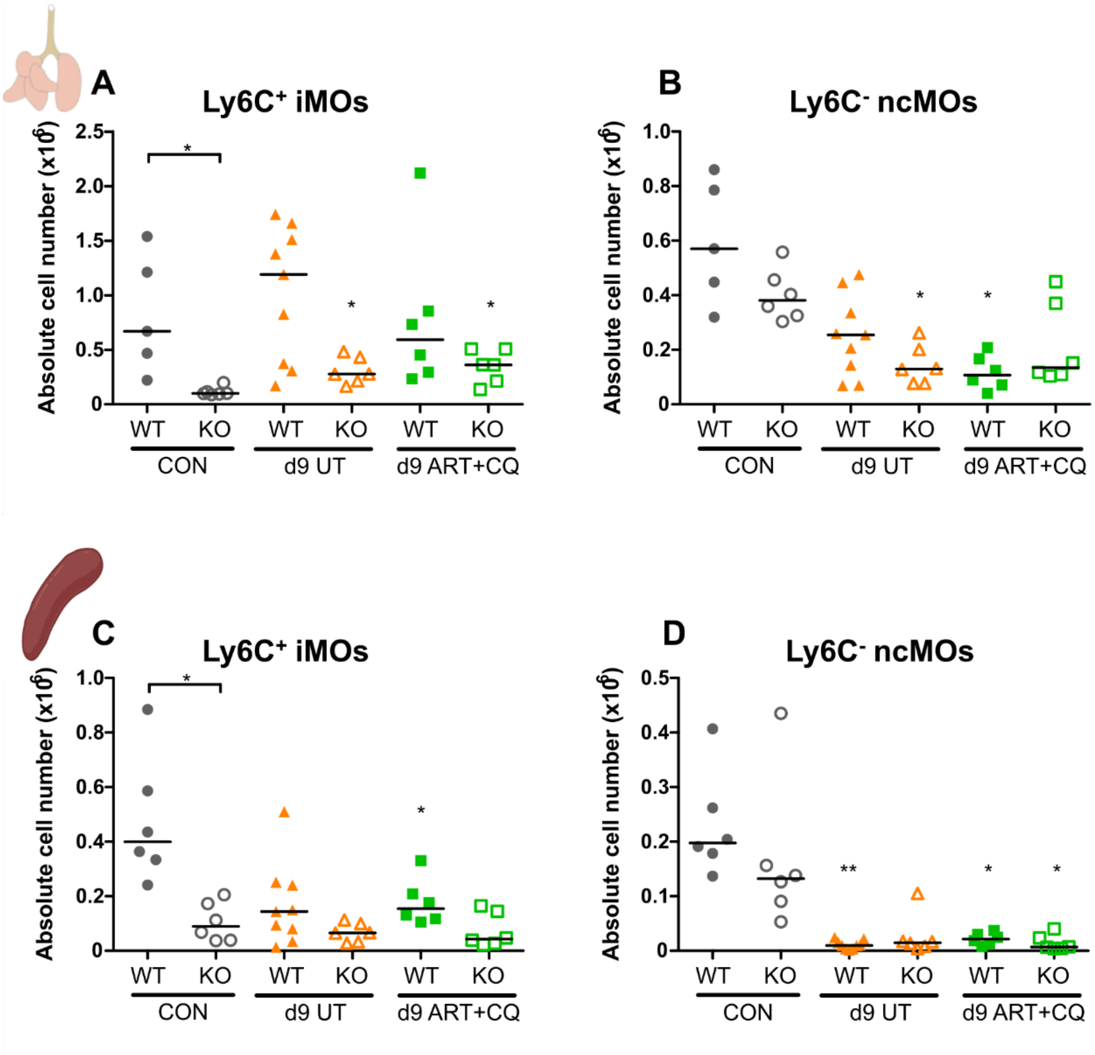

Supplementary Figure 8. CCR2 gene knock-out induced a decreased trend in pulmonary inflammatory monocytes at 9 days p.i.

CCR2 WT and CCR2 KO C57BL/6 mice were infected with *Pb*NK65. Mice were injected daily from 8 until 12 days p.i. with 10 mg/kg ART + 30 mg/kg CQ. Mice were dissected at the indicated days p.i. Leukocytes were isolated from the lungs according to protocol 2 and from the spleen and flow cytometry was performed. (A-D) The absolute numbers of Ly6C$^+$ iMOs (A,C) and Ly6C$^-$ ncMOs (B,D) present in the lungs (A,B) and spleen (C,D) were calculated. Compilation of two experiments, except ART+ CQ CCR2 WT and KO group only one experiment. Each symbol represents data of an individual mouse. n=5 for CON CCR2 WT, n=6 for CON CCR2 KO, n=9 for UT CCR2 WT, n=6 for UT CCR2 KO, n=6 for ART+CQ CCR2 WT, n=6 for ART+CQ CCR2 KO.

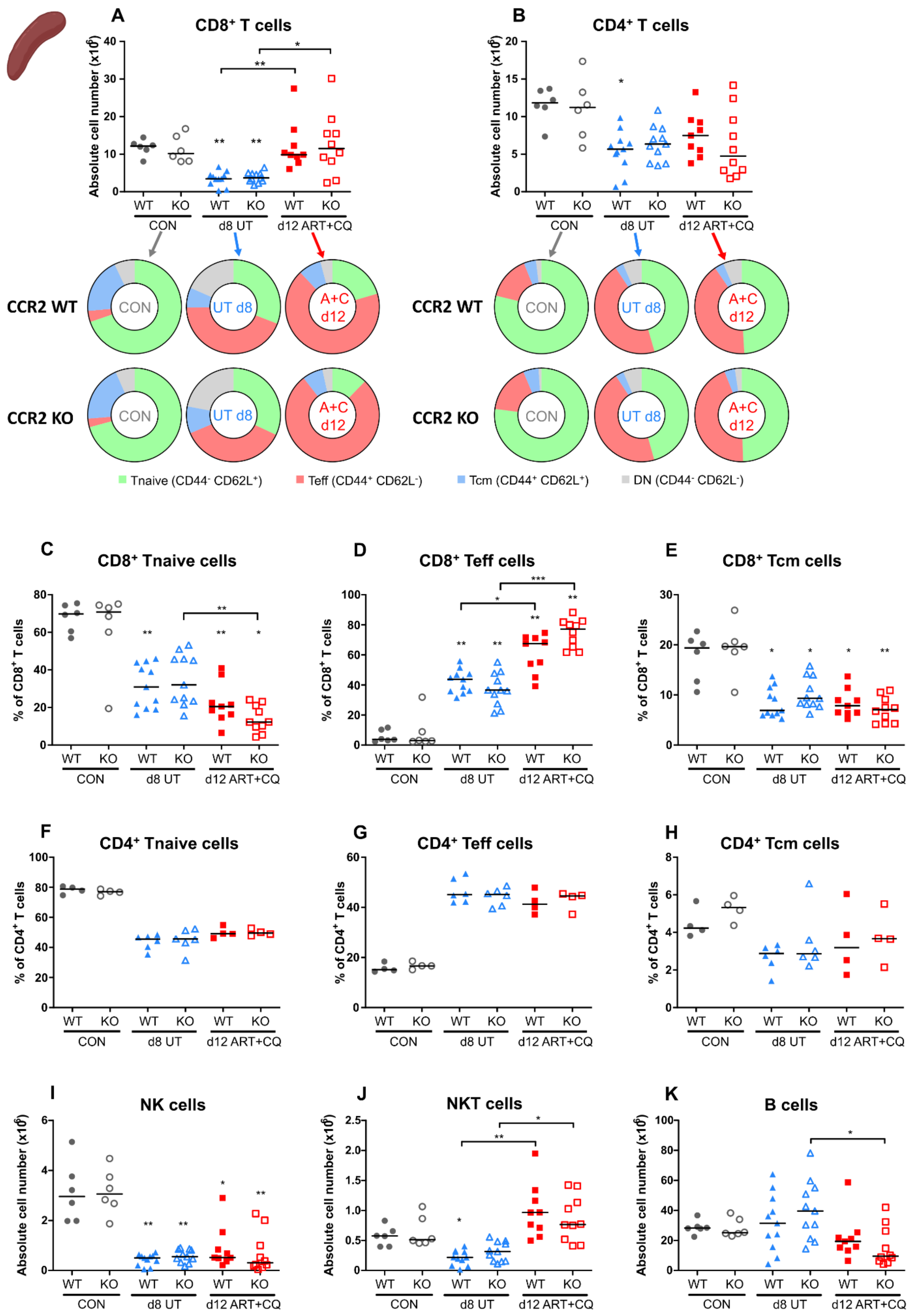
A
CD8+ T cells
B
CD4+ T cells
Absolute cell number (x10^6)
WT KO WT KO WT KO
CON d8 UT d12 ART+CQ
CCR2 WT
CCR2 KO
CON
UT d8
A+C d12
Tnaive (CD44- CD62L+)
Teff (CD44+ CD62L-)
Tcm (CD44+ CD62L+)
DN (CD44- CD62L-)
C
CD8+ Tnaive cells
D
CD8+ Teff cells
E
CD8+ Tcm cells
% of CD8+ T cells
F
CD4+ Tnaive cells
G
CD4+ Teff cells
H
CD4+ Tcm cells
% of CD4+ T cells
I
NK cells
J
NKT cells
K
B cells

Supplementary Figure 9. No effect of CCR2 gene knock-out was observed on the number of splenic lymphocytes.

CCR2 WT and CCR2 KO C57BL/6 mice were infected with *Pb*NK65. Mice were injected daily from 8 until 12 days p.i. with 10 mg/kg ART + 30 mg/kg CQ. Mice were dissected at the indicated days p.i. Leukocytes were isolated from the spleen and flow cytometry was performed. (A-H) The absolute numbers of $CD8^+$ T cells ($CD45^+$ $CD3^+$ $NK1.1^-$ $CD8^+$) and $CD4^+$ T cells ($CD45^+$ $CD3^+$ $NK1.1^-$ $CD4^+$) and the percentages of Tnaive ($CD44^-$ $CD62L^+$), Teff ($CD44^+$ $CD62L^-$) and Tcm ($CD44^+$ $CD62L^+$) of the total cell population are shown. (I-K) The absolute numbers of NK cells ($CD45^+$ $CD3^-$ $NK1.1^+$), NKT cells ($CD45^+$ $CD3^+$ $NK1.1^+$) and B cells ($CD45^+$ $CD3^-$ $NK1.1^-$ $B220^+$) were calculated. Compilation of two experiments, except activation of $CD4^+$ T cells (F-H) only one experiment. Each symbol represents data of an individual mouse. n=4-6 for CON CCR2 WT, n=4-6 for CON CCR2 KO, n=6-11 for UT CCR2 WT, n=6-11 for UT CCR2 KO, n=4-9 for ART+CQ CCR2 WT, n=4-10 for ART+CQ CCR2 KO.

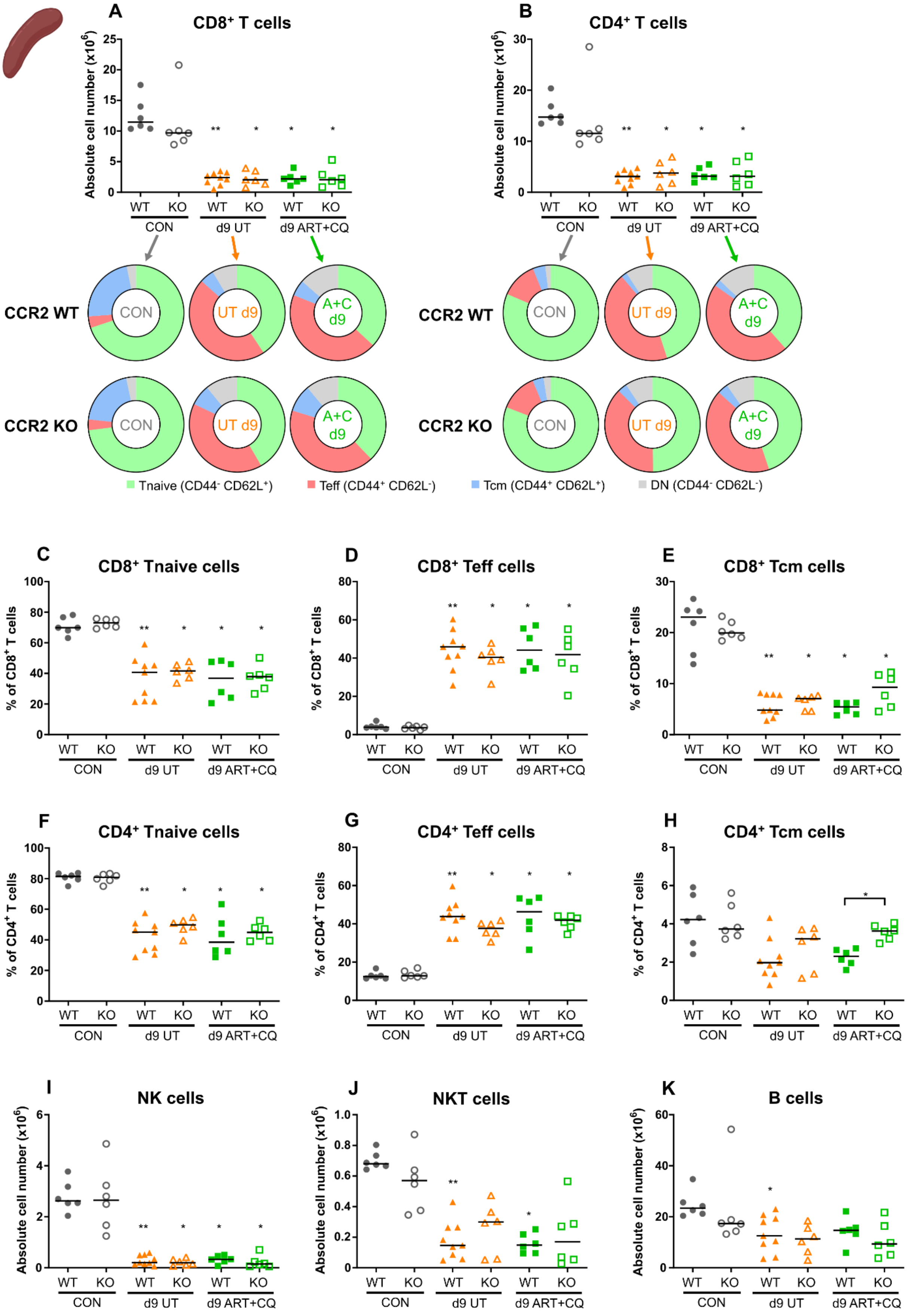
A
CD8⁺ T cells
Absolute cell number (x10⁶)
B
CD4⁺ T cells
WT KO WT KO WT KO
CON
d9 UT
d9 ART+CQ
CCR2 WT
CCR2 KO
CON
UT d9
A+C d9
Tnaive (CD44⁻ CD62L⁺)
Teff (CD44⁺ CD62L⁻)
Tcm (CD44⁺ CD62L⁺)
DN (CD44⁻ CD62L⁻)
C
CD8⁺ Tnaive cells
D
CD8⁺ Teff cells
E
CD8⁺ Tcm cells
% of CD8⁺ T cells
F
CD4⁺ Tnaive cells
G
CD4⁺ Teff cells
H
CD4⁺ Tcm cells
% of CD4⁺ T cells
I
NK cells
J
NKT cells
K
B cells

Supplementary Figure 10. No effect of CCR2 gene knock-out was observed on the number of splenic lymphocytes at 9 days p.i.

CCR2 WT and CCR2 KO C57BL/6 mice were infected with *Pb*NK65. Mice were injected at 8 days p.i. with 10 mg/kg ART + 30 mg/kg CQ. Mice were dissected at the indicated days p.i. Leukocytes were isolated from the spleen and flow cytometry was performed. (A-H) The absolute numbers of $CD8^+$ T cells ($CD45^+$ $CD3^+$ $NK1.1^-$ $CD8^+$) and $CD4^+$ T cells ($CD45^+$ $CD3^+$ $NK1.1^-$ $CD4^+$) and the percentages of Tnaive ($CD44^-$ $CD62L^+$), Teff ($CD44^+$ $CD62L^-$) and Tcm ($CD44^+$ $CD62L^+$) of the total cell population are shown. (I-K) The absolute numbers of NK cells ($CD45^+$ $CD3^-$ $NK1.1^+$), NKT cells ($CD45^+$ $CD3^+$ $NK1.1^+$) and B cells ($CD45^+$ $CD3^-$ $NK1.1^-$ $B220^+$) were calculated. Compilation of two experiments, except ART+ CQ CCR2 WT and KO group only one experiment. Each symbol represents data of an individual mouse. n=6 for CON CCR2 WT, n=6 for CON CCR2 KO, n=9 for UT CCR2 WT, n=6 for UT CCR2 KO, n=6 for ART+CQ CCR2 WT, n=6 for ART+CQ CCR2 KO.

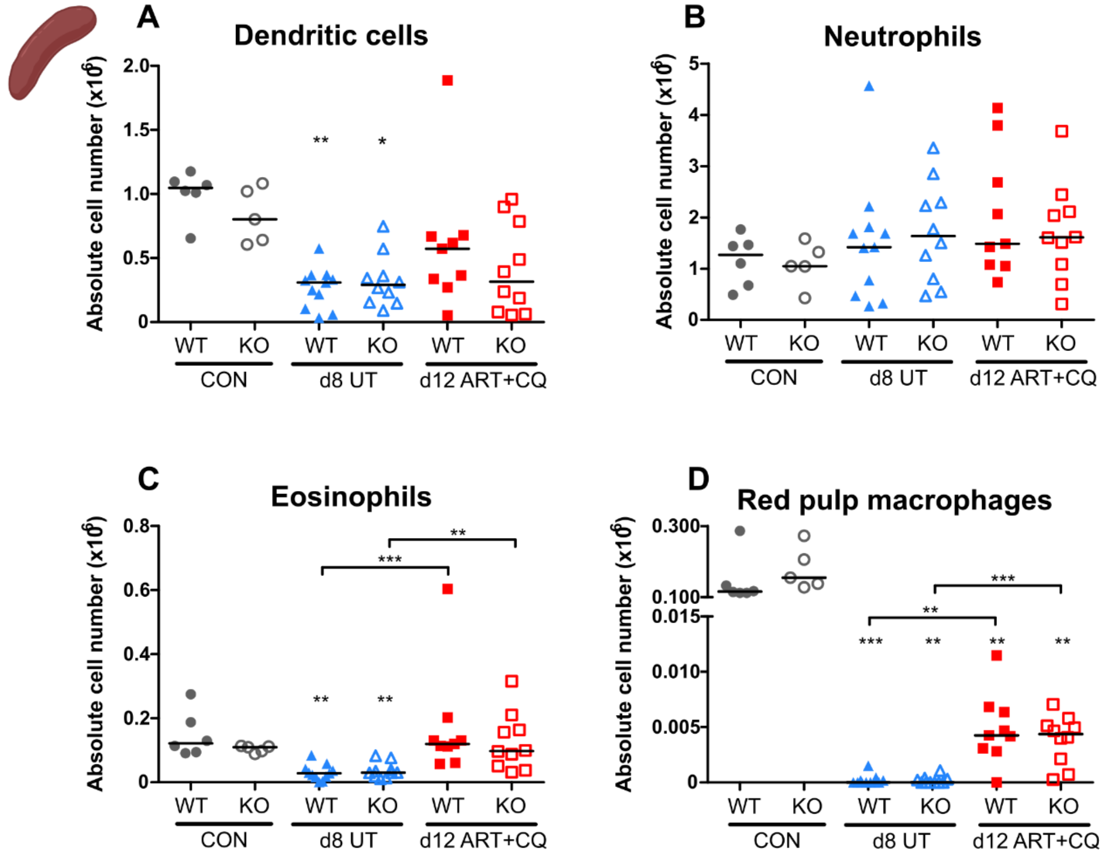

Supplementary Figure 11. CCR2 gene knock-out had no effect on the myeloid cell populations in the spleen.

CCR2 WT and CCR2 KO C57BL/6 mice were infected with *Pb*NK65. Mice were injected daily from 8 until 12 days p.i. with 10 mg/kg ART + 30 mg/kg CQ. Mice were dissected at the indicated days p.i. Leukocytes were isolated from the spleen and flow cytometry was performed. (A-D) The absolute numbers of dendritic cells ($CD45^+$ Lin^- MHC-II^+ $CD11c^+$), neutrophils ($CD45^+$ Lin^- $CD11b^+$ $Ly6G^+$), eosinophils ($CD45^+$ Lin^- $CD11b^+$ $Ly6G^-$ $SiglecF^+$) and red pulp macrophages ($CD45^+$ Lin^- MHC-II^- $CD11c^-$ $CD11b^-$ $F4/80^+$) present in the spleen were calculated. Compilation of two experiments. Each symbol represents data of an individual mouse. n=6 for CON CCR2 WT, n=5 for CON CCR2 KO, n=11 for UT CCR2 WT, n=10 for UT CCR2 KO, n=9 for ART+CQ CCR2 WT, n=10 for ART+CQ CCR2 KO.

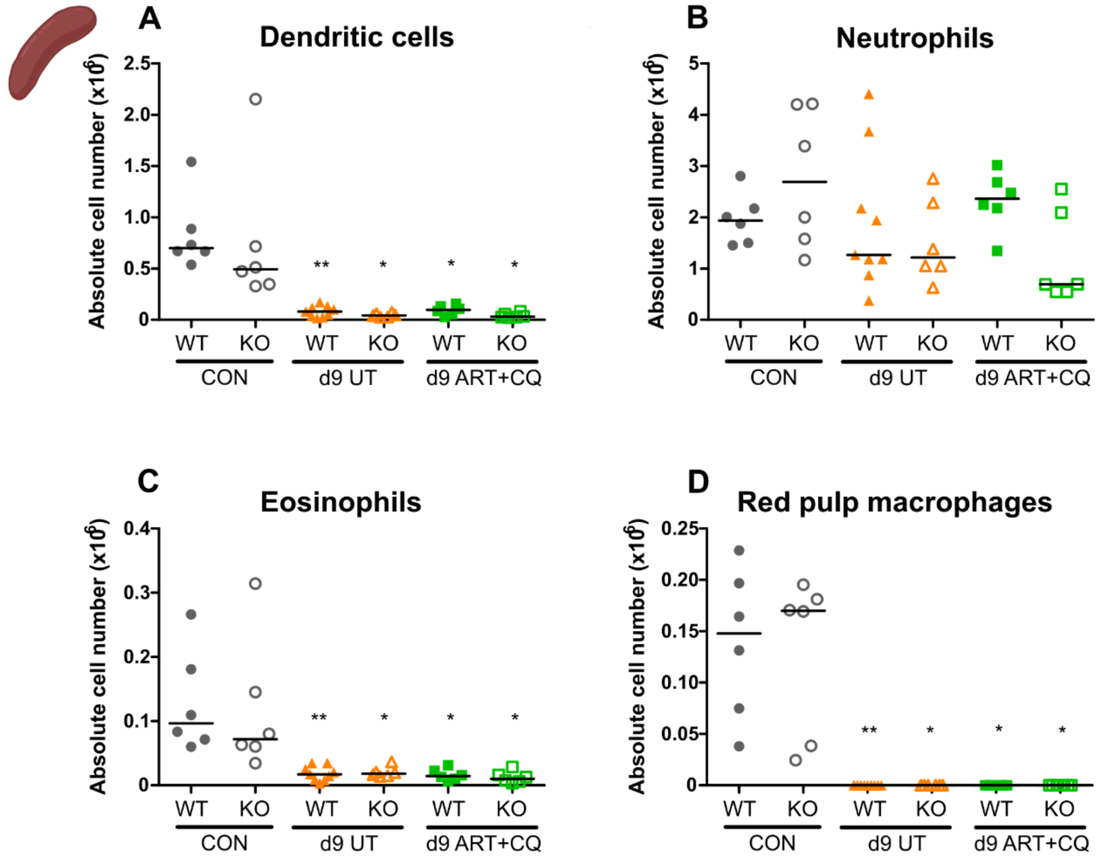

Supplementary Figure 12. CCR2 gene knock-out had no effect on the number of myeloid cells present at 9 days p.i. in the spleen.

CCR2 WT and CCR2 KO C57BL/6 mice were infected with *Pb*NK65. Mice were injected at 8 days p.i. with 10 mg/kg ART + 30 mg/kg CQ. Mice were dissected at the indicated days p.i. Leukocytes were isolated from the spleen and flow cytometry was performed. (A-D) The absolute numbers of dendritic cells ($CD45^+$ Lin^- $MHC\text{-}II^+$ $CD11c^+$), neutrophils ($CD45^+$ Lin^- $CD11b^+$ $Ly6G^+$), eosinophils ($CD45^+$ Lin^- $CD11b^+$ $Ly6G^-$ $SiglecF^+$) and red pulp macrophages ($CD45^+$ Lin^- $MHC\text{-}II^-$ $CD11c^-$ $CD11b^-$ $F4/80^+$) present in the spleen were calculated. Compilation of two experiments, except ART+ CQ CCR2 WT and KO group only one experiment. Each symbol represents data of an individual mouse. n=6 for CON CCR2 WT, n=6 for CON CCR2 KO, n=9 for UT CCR2 WT, n=6 for UT CCR2 KO, n=6 for ART+CQ CCR2 WT, n=6 for ART+CQ CCR2 KO.

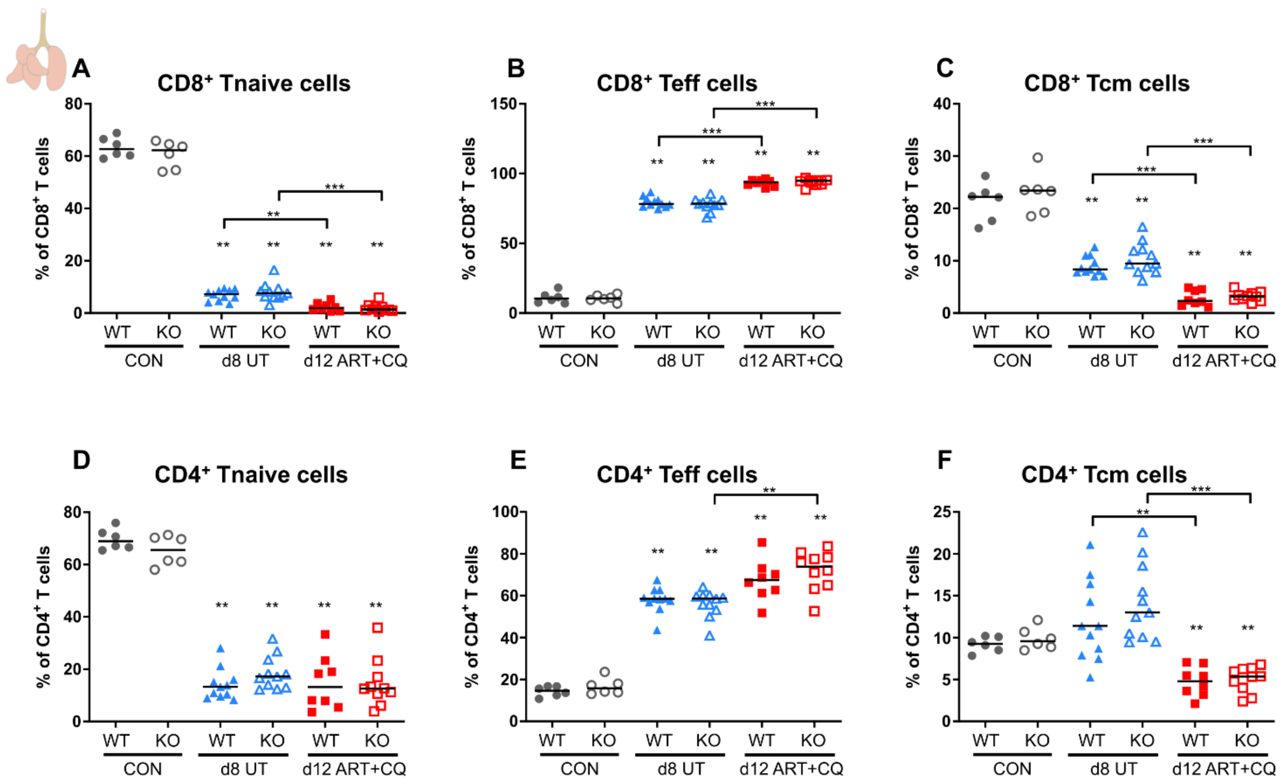

Supplementary Figure 13. Activation status of pulmonary CD4⁺ and CD8⁺ T cells was not affected in CCR2 KO mice.

CCR2 WT and CCR2 KO C57BL/6 mice were infected with *Pb*NK65. Mice were injected daily from 8 until 12 days p.i. with 10 mg/kg ART + 30 mg/kg CQ. Mice were dissected at the indicated days p.i. Leukocytes were isolated from the lungs according to protocol 2 and flow cytometry was performed. (A-C) The percentages of Tnaive ($CD44^- CD62L^+$), Teff ($CD44^+ CD62L^-$) and Tcm ($CD44^+ CD62L^+$) of the total $CD8^+$ T cell population are shown. (D-F) The percentages of Tnaive ($CD44^- CD62L^+$), Teff ($CD44^+ CD62L^-$) and Tcm ($CD44^+ CD62L^+$) of the total $CD4^+$ T cell population are shown. Compilation of two experiments. Each symbol represents data of an individual mouse. n=6 for CON CCR2 WT, n=6 for CON CCR2 KO, n=11 for UT CCR2 WT, n=11 for UT CCR2 KO, n=8 for ART+CQ CCR2 WT, n=10 for ART+CQ CCR2 KO.

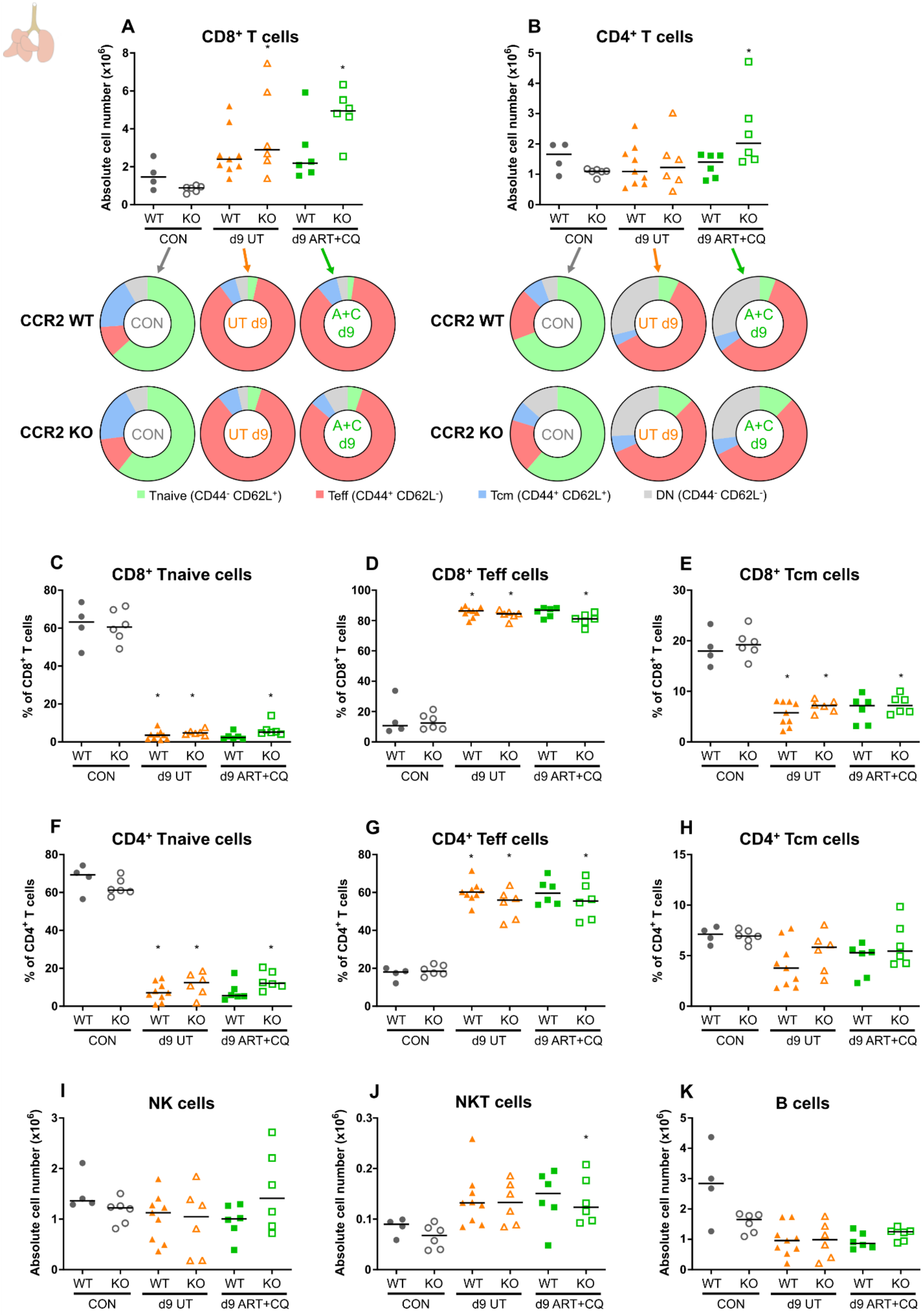
A
CD8⁺ T cells
Absolute cell number (x10⁶)
WT KO WT KO WT KO
CON
d9 UT
d9 ART+CQ
B
CD4⁺ T cells
CCR2 WT
CCR2 KO
CON
UT d9
A+C d9
Tnaive (CD44⁻ CD62L⁺)
Teff (CD44⁺ CD62L⁻)
Tcm (CD44⁺ CD62L⁺)
DN (CD44⁻ CD62L⁻)
C
CD8⁺ Tnaive cells
% of CD8⁺ T cells
D
CD8⁺ Teff cells
E
CD8⁺ Tcm cells
F
CD4⁺ Tnaive cells
% of CD4⁺ T cells
G
CD4⁺ Teff cells
H
CD4⁺ Tcm cells
I
NK cells
J
NKT cells
K
B cells

Supplementary Figure 14. No effect of CCR2 gene knock-out was observed on the number of pulmonary lymphocytes at 9 days p.i.

CCR2 WT and CCR2 KO C57BL/6 mice were infected with *Pb*NK65. Mice were injected at 8 days p.i. with 10 mg/kg ART + 30 mg/kg CQ. Mice were dissected at the indicated days p.i. Leukocytes were isolated from the lungs according to protocol 2 and flow cytometry was performed. (A-H) The absolute numbers of $CD8^+$ T cells ($CD45^+$ $CD3^+$ $NK1.1^-$ $CD8^+$) and $CD4^+$ T cells ($CD45^+$ $CD3^+$ $NK1.1^-$ $CD4^+$) and the percentages of Tnaive ($CD44^-$ $CD62L^+$), Teff ($CD44^+$ $CD62L^-$) and Tcm ($CD44^+$ $CD62L^+$) of the total cell population are shown. (I-K) The absolute numbers of NK cells ($CD45^+$ $CD3^-$ $NK1.1^+$), NKT cells ($CD45^+$ $CD3^+$ $NK1.1^+$) and B cells ($CD45^+$ $CD3^-$ $NK1.1^-$ $B220^+$) were calculated. Compilation of two experiments, except ART+ CQ CCR2 WT and KO group only one experiment. Each symbol represents data of an individual mouse. n=4 for CON CCR2 WT, n=6 for CON CCR2 KO, n=9 for UT CCR2 WT, n=6 for UT CCR2 KO, n=6 for ART+CQ CCR2 WT, n=6 for ART+CQ CCR2 KO.

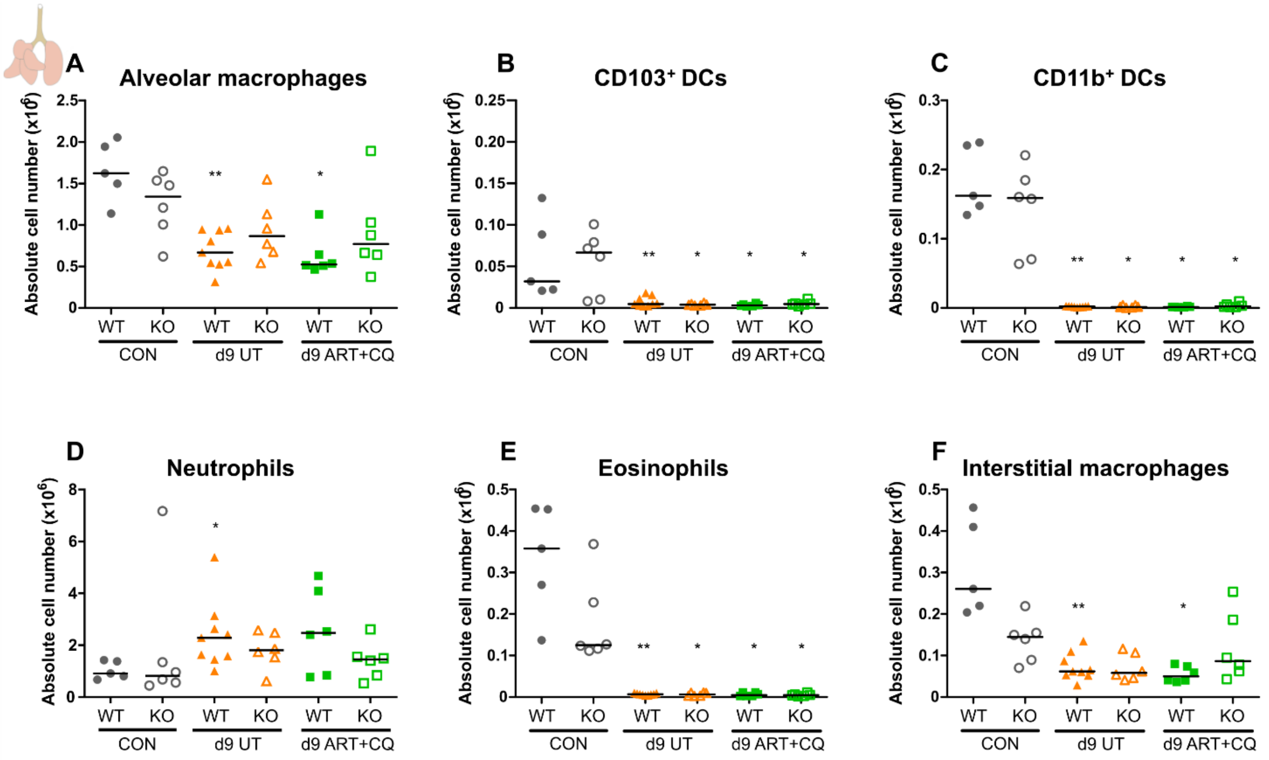

Supplementary Figure 15. CCR2 gene knock-out had no effect on the number of myeloid cells present at 9 days p.i. in the lungs.

CCR2 WT and CCR2 KO C57BL/6 mice were infected with *Pb*NK65. Mice were injected at 8 days p.i. with 10 mg/kg ART + 30 mg/kg CQ. Mice were dissected at the indicated days p.i. Leukocytes were isolated from the lungs according to protocol 2 and flow cytometry was performed. (A-F) The absolute numbers of alveolar macrophages (AM; $CD45^+$ $SiglecF^+$ $CD11b^{int}$ $CD11c^+$), $CD103^+$ dendritic cells ($CD103^+$ DCs; $CD45^+$ Lin^- $SiglecF^-$ MHC-II^+ $CD11c^+$ $CD103^+$ $CD11b^-$), $CD11b^+$ dendritic cells ($CD11b^+$ DCs; $CD45^+$ Lin^- $SiglecF^-$ MHC-II^+ $CD11c^+$ $CD103^-$ $CD11b^+$ $CD24^+$ $CD64^-$), neutrophils (Neutros; $CD45^+$ Lin^- $CD11b^+$ $Ly6G^+$), eosinophils (Eos; $CD45^+$ $CD11b^+$ $SiglecF^+$ $CD11c^-$) and interstitial macrophages (IM; $CD45^+$ Lin^- $CD11b^{hi}$ MHC-II^+ $CD64^+$ $CD24^-$) present in the lungs were calculated. Compilation of two experiments, except ART+ CQ CCR2 WT and KO group only one experiment. Each symbol represents data of an individual mouse. n=5 for CON CCR2 WT, n=6 for CON CCR2 KO, n=9 for UT CCR2 WT, n=6 for UT CCR2 KO, n=6 for ART+CQ CCR2 WT, n=6 for ART+CQ CCR2 KO.

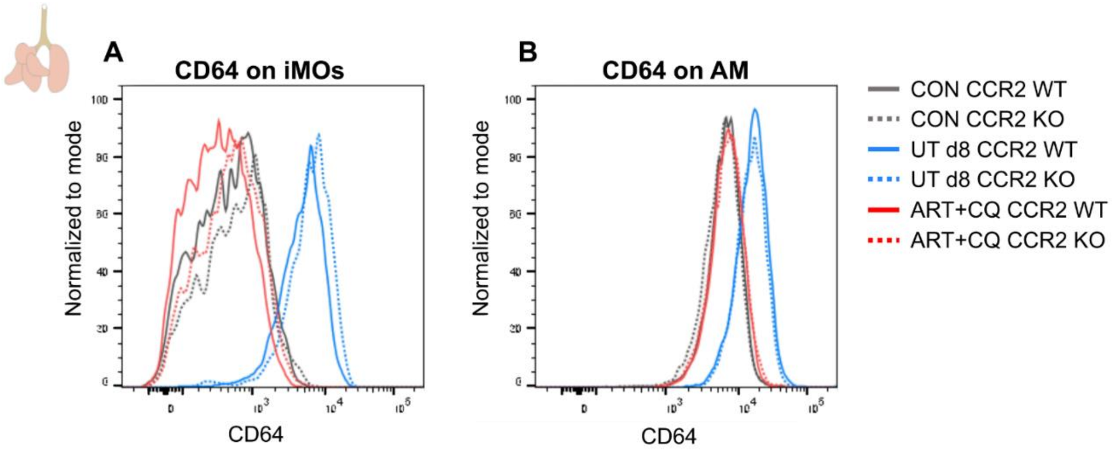

Supplementary Figure 16. CD64 expression by iMOs and AM during resolution in CCR2 KO and WT mice.

CCR2 WT and CCR2 KO C57BL/6 mice were infected with *Pb*NK65. Mice were injected daily from 8 until 12 days p.i. with 10 mg/kg ART + 30 mg/kg CQ. Mice were dissected at the indicated days p.i. Leukocytes were isolated from the lungs according to protocol 2 and flow cytometry was performed. Representative histograms of the expression of CD64 on inflammatory monocytes (iMOs (A); $CD45^{+}$ Lin^{-} $CD11b^{hi}$ MHC-II^{-} $Ly6C^{+}$) and alveolar macrophages (AM (B); $CD45^{+}$ $SiglecF^{+}$ $CD11b^{int}$ $CD11c^{+}$) are shown.
