# Supplementary material for: CCR2 Is Dispensable for Disease Resolution but Required for the Restoration of Leukocyte Homeostasis Upon Experimental Malaria-Associated Acute Respiratory Distress Syndrome
Source: Front Immunol. 2021 Feb 16;11:628643. doi: 10.3389/fimmu.2020.628643 (PMC7921736; doi:10.3389/fimmu.2020.628643)
Supplement: Supplementary file 3 [file Table_1.docx]

**Supplementary table 1. Primers used for RT-qPCR**

| **Gene** | **Full protein name** | **Exon location** | **Fluorescent dye** | **Catalog number primer** |
| --- | --- | --- | --- | --- |
| 18S | 18S ribosomal RNA | 1-1 | FAM^TM^ | Hs.PT.39a.22214856.g |
| IFN-γ | Interferon gamma | 1-2 | FAM^TM^ | Mm.PT.58.41769240 |
| TNF-α | Tumor necrosis factor alpha | 2-4 | FAM^TM^ | Mm.PT.58.12575861 |
| CCL2  (MCP-1) | Monocyte chemoattractant protein-1 | 1-3 | FAM^TM^ | Mm.PT.58.42151692 |
| CXCL10  (IP-10) | IFN-γ-inducible protein-10 | 1-2 | FAM^TM^ | Mm.PT.58.43575827 |
| IL-10 | Interleukin-10 | 3-5 | FAM^TM^ | Mm.PT.58.13531087 |
