# Supplementary material for: CCR2 Is Dispensable for Disease Resolution but Required for the Restoration of Leukocyte Homeostasis Upon Experimental Malaria-Associated Acute Respiratory Distress Syndrome
Source: Front Immunol. 2021 Feb 16;11:628643. doi: 10.3389/fimmu.2020.628643 (PMC7921736; doi:10.3389/fimmu.2020.628643)
Supplement: Supplementary file 4 [file Table_2.docx]

**Supplementary Table 2. Antibodies used for flow cytometry**

| **Antigen** | **Clone** | **Fluorophore** | **Company** |
| --- | --- | --- | --- |
| **Lymphoid panel (100 000 cells read)** | | | |
| CD3 | 145-2C11 | FITC | eBioscience |
| CD8 | 53-6.7 | PerCP-Cy5.5 | eBioscience |
| CD44 | IM7 | PE | Biolegend |
| NK1.1 | PK136 | PE-Cy7 | eBioscience |
| CD4 | RM4-5 | APC-eFluor 780 | eBioscience |
| CD62L | MEL-14 | APC | eBioscience |
| B220 | RA3-6B2 | BV786 | BD |
| CD45 | 30-F11 | BUV395 | BD |
| Live/dead |  | Zombie Aqua™ Fixable Viability Kit | Biolegend |
| **Myeloid panel (200 000 cells read)** | | | |
| CD45 | 30-F11 | FITC | Biolegend |
| CD103 | 2E7 | PerCP-eFluor 710 | eBioscience |
| CD11c | N418 | PE-Cy7 | Biolegend |
| CD64 | X54-5/7.1 | PE | Biolegend |
| CD24 | M1/69 | PE-CF594 | Biolegend |
| SiglecF | 1RNM44N | eFluor 660 | eBioscience |
| Ly6G | 1A8 | Alexa fluor 700 | BD |
| Ly6C | AL-21 | APC-Cy7 | BD |
| CD11b | M1/70 | eFluor 450 or PerCP-Cy5.5 | eBioscience |
| MHC-II | M5/114 | Horizon v500 | BD |
| CD3 | 17A2 | BV650 | Biolegend |
| CD19 | 6D5 | BV650 | Biolegend |
| NK1.1 | PK136 | BV650 | Biolegend |
| Live/dead |  | Zombie UV™ Fixable Viability Kit | Biolegend |
| **CCR2 panel (200 000 cells read)** | | | |
| Ly6C | HK1.4 | FITC | Biolegend |
| CD11c | N418 | PE-Cy7 | Biolegend |
| SiglecF | E50-2440 | PE-CF594 | BD |
| CCR2 | SA203G11 | APC | Biolegend |
| Ly6G | 1A8 | Alexa fluor 700 | BD |
| CD11b | M1/70 | eFluor 450 | eBioscience |
| CD3 | 17A2 | BV650 | Biolegend |
| CD19 | 6D5 | BV650 | Biolegend |
| NK1.1 | PK136 | BV650 | Biolegend |
| Live/dead |  | Zombie UV™ Fixable Viability Kit | Biolegend |
